# Mathematical modeling of solid cancer growth with angiogenesis

**DOI:** 10.1186/1742-4682-9-2

**Published:** 2012-02-02

**Authors:** Hyun M Yang

**Affiliations:** 1 UNICAMP - IMECC - DMA, Praça Sérgio Buarque de Holanda, 651, CEP: 13083-859, Campinas, SP, Brazil

## Abstract

**Background:**

Cancer arises when within a single cell multiple malfunctions of control systems occur, which are, broadly, the system that promote cell growth and the system that protect against erratic growth. Additional systems within the cell must be corrupted so that a cancer cell, to form a mass of any real size, produces substances that promote the growth of new blood vessels. Multiple mutations are required before a normal cell can become a cancer cell by corruption of multiple growth-promoting systems.

**Methods:**

We develop a simple mathematical model to describe the solid cancer growth dynamics inducing angiogenesis in the absence of cancer controlling mechanisms.

**Results:**

The initial conditions supplied to the dynamical system consist of a perturbation in form of pulse: The origin of cancer cells from normal cells of an organ of human body. Thresholds of interacting parameters were obtained from the steady states analysis. The existence of two equilibrium points determine the strong dependency of dynamical trajectories on the initial conditions. The thresholds can be used to control cancer.

**Conclusions:**

Cancer can be settled in an organ if the following combination matches: better fitness of cancer cells, decrease in the efficiency of the repairing systems, increase in the capacity of sprouting from existing vascularization, and higher capacity of mounting up new vascularization. However, we show that cancer is rarely induced in organs (or tissues) displaying an efficient (numerically and functionally) reparative or regenerative mechanism.

## Background

A total of 562, 875 cancer deaths were recorded in the United States in 2007, and it is estimated that approximately 570, 000 died from cancer in 2010. The overall estimate of approximately 1.53 million new cases does not include carcinoma *in situ *of any site except urinary bladder, nor does it include basal cell and squamous cell cancers of the skin. Greater than 2 million unreported cases of basal cell and squamous cell skin cancer, approximately 54, 010 cases of breast carcinoma *in situ*, and 46, 770 cases of melanoma *in situ *are expected to be newly diagnosed in 2010 [1]. In the world, the estimate for 1990 suggested a total of 8.1 million new cases, divided almost exactly between developed and developing countries, and 5.2 million cancer deaths, about 55% of which occurred in developing countries [2].

Cells of some organs, as the heart, stop proliferation after reaching their size, but others, like skin cells and cells that line body cavities, must proliferate almost continuously to replenish cells that are lost. Cancer arises when within a single cell multiple malfunctions of control systems occur, which are, broadly, the system that promote cell growth and the system that protect against erratic growth (for instance, tumor suppressor gene p53, which name comes from protein of molecular weight **53**,000, and RB, from retinoblastoma). Additional systems within the cell must be corrupted so that a cancer cell, to form a mass of any real size, produces substances (such as VEGF - Vascular Endothelial Growth Factor) that promote the growth of new blood vessels. Cellular growth-control systems can be corrupted either when cellular genes are mutated or when proteins produced during a viral infection interfere with control system function. Multiple mutations are required before a normal cell can become a cancer cell by corruption of multiple (about five) growth-promoting systems [3].

With respect to the control systems that protect against cancer, they are classified in two general types: systems that prevent mutations, and systems that deal with mutations once they occurred. For instance, there are mechanisms in cells that can convert toxic by-products of cellular metabolism or carcinogenic substances from the environment into harmless chemicals, preventing DNA damage. In addition, cells have a number of different systems that can detect damaged DNA and fix it, avoiding mutations. However, if the DNA of a cell has been damaged that repair would be impossible, there are systems which trigger the cell to die. Hence, even though there are trillions of cells, only one in three humans will get cancer during his lifetime, and cancer is mainly a disease of the elderly [3].

In order to develop effective treatments, it is important to identify the mechanisms to controlling cancer growth, how they interact, and how they can most easily be manipulated to eradicate (or manage) the disease. Aiming to gain such insight, it is usually necessary to perform large numbers of time-consuming and intricate experiments - but not always. Through the development and solution of mathematical models that describe different aspects of solid tumor growth, applied mathematics has the potential to prevent excessive experimentation and also to provide biologists with complementary and valuable insight into the mechanisms that may control the development of solid tumors [4]. In [5] it is provided a concise history of the study of tumor growth, discussing some of the most influential mathematical models and their relationship to experimental studies, and illustrating how the field of cancer research has evolved due to these interactions between theoretical and experimental approaches.

In this paper we develop a mathematical model to understand the solid cancer growth inducing angiogenesis. We determine the steady states of the model and the stability of the equilibrium points are performed. We simulate the model to assess the appearance of cancer. Treatments aiming the control of cancer are not dealt here (see [6] and [7] for tumor control).

## Methods

Our objective is the development of a simple mathematical model to describe the cancer growth dynamics inducing angiogenesis. The model does not include important physiological processes like the diffusion of oxygen into a solid where it is consumed by metabolic processes, the outward diffusion of lactic acid from a solid which produces it by metabolic processes and the diffusion of oxygen away from a blood vessel into a region with an oxygen debt. First, we describe an organ of human body free of cancer and, then, cancer cells appear in this organ.

The normal cells of an organ (the concentration at time *t *is designed as *C*) are feed by an existing vasculature formed by endothelial cells (the concentration at time *t *is designed as *E*). Adult endothelial cells and cells of an organ of human body are normally quiescent apart from certain developmental processes (e.g., embryogenesis), and proliferation of these cells aims to replenish losses (for instance, cells dying due to aging and wound). The normal cells and surrounding vascular networks (epithelial cells) are governed by the logistic growth, with intrinsic growth rates *α*_1 _and *α*_2_, respectively, and they are under mortality rates *μ*_1 _and *μ*_2_, respectively. Normal cells produce substances to promote the growth of blood vessels in a suitable network to attend their needs. The extension of vascularization (blood vessels network) depends on the size and function of the organ, and conversely, the blood vessels network determines the activity of the organ. In other words, the size of an organ could be determined by the surrounding network of blood vessels, that is, *k*_1_(*E*), as *k*_2 _depends on *C, k*_2_(*C*), where *k*_1 _and *k*_2 _are the carrying capacities of the organ (normal cells) and the extension of blood vessels network (epithelial cells), respectively. In a cancer free state, normal cells (*C*) and blood vessels (*E*) are in a steady state, where the amount of normal cells depends on the feeding network, as well as the amount of epithelial cells depends on the size and function of organs that must feed.

Let us suppose that a series of accumulating mutations in normal cells (one single cell may be sufficient) corrupted systems that promote cell growth and that protect against erratic growth. Also systems that control the production of substances which induce new blood vessels are corrupted in the cancer cells. These sequential events initiate a rapid growth of cancer due to angiogenesis. Angiogenesis is the process by which new blood vessels develop from an existing vasculature, through endothelial cell sprouting, proliferation, and fusion [8]. The angiogenesis links the relatively harmless avascular and the potentially fatal vascular growth phases of the tumor [9].

Let us consider what occurs in cancer growth: (1) there is an initial amount of cancer cells (the model does not deal with the origin of cancer cells) appearing in a completely health organ; (2) these cells induce the formation of pre-vascular cells from existing vascular network; (3) after a period of time, the formation of new blood vessels is initiated; and (4) both normal and cancer cells compete for nutrients and space (proteins, oxygen, etc.).

For cancer cells (the concentration at time *t *is designed as *T*), the growth, which is likely to be limited by energetic constraints, is limited by the carrying capacity *k*_3 _and the surrounding new vessels originated by angiogenesis (the concentration at time *t *is designed as *A*). The new vessels, represented by angiogenesis cells *A*, have growth depending on tumor cells *T *and are limited by available space (*k*_4_). The growth of blood vessels (*E *and *A*) are constrained by physical restrictions, even they are never subject to either oxygen or nutrient deprivation. We assume the existence of an early stage of angiogenesis, called pre-angiogenesis (the concentration of cells at time *t *is designed as *P*). This stage corresponds to the release by tumoral cells of VEGF, which starts to diffuse into the surrounding tissue and approach the endothelial cells of nearby blood vessels. Endothelial cells subsequently respond to the VEGF concentration gradient by forming sprouts. From these sprouts, there occur migration and proliferation toward the tumor, and these new vessels are called as angiogenesis (endothelial cells *A*).

The tumoral cells induce new vascular network from the existing one to feed cancer cells. The pre-angiogenesis cells are formed by the mass action law [10] at a constant rate *γ*, the rate of sprouts formation, from which new vascularizations occur resulting in angiogenesis cells after an average period of time *δ*^-1^, where *δ *is transfer rate from pre-angiogenesis to angiogenesis cells. The cancer, pre-angiogenesis and angiogenesis cells are under the mortality rates *μ*_3_, *μ*_4 _and *μ*_5_, respectively. The cancer and angiogenesis cells are governed by the logistic growth: intrinsic growth *α*_3 _and *ε*, which depend respectively on angiogenesis and cancer cells, and carrying capacities *k*_3 _and *k*_4_, respectively. Cancer cells can also grow logistically using existing vascularization at rate $\alpha_{3}^{\prime }$. The rates at which normal and cancer cells compete themselves for resources and space are $\beta_{1}^{\prime }$ and $\beta_{2}^{\prime }$. Both parameters also take into account effects of the environment.

Pre-angiogenesis cells deserve some words. New blood vessels to nourish cancer cells must sprout from pre-existing blood vessels. However, there is an elapse of time from the appearance of sprouts until the complete formation of new blood vessels that effectively feed cancer cells. By doing this, we take into account a time-delay between the formation of new network of blood vessels (angiogenesis cells *A*) and tumor growth. Notice that the probability of sproutings becoming angiogenesis cells is *δ*/(*δ *+ *μ*_4_). If we let *δ *→ ∞, this phase is negligible (probability one).

Let us consider the normal cells *C *taking into account the above descriptions. In the absence of cancer cells *T*, normal cells obey $\frac{d}{dt}C=\alpha_{1}C\left[1-\frac{C}{k_{1}\left(E\right)}\right]-\mu_{1}C$. The cancer cells influence negatively normal cells, and this term is described by $\frac{\alpha_{1}C{\beta^{\prime }}_{1}T}{k_{1}\left(E\right)}$ with negative signal. Due to mutation, some normal cells become cancer cells, which can occur continuously. However, we will assume that the mutations occur instantaneously at a given time (cancer cells grow exponentially in the beginning, hence further mutations, being in small amount, can be disregarded), when an amount of normal cells are transferred to cancer cells compartment. For other types of cells, the dynamical equations can be obtained similarly.

Based on the above definitions of variables and model's parameters, the dynamics of the cancer growth is described by the following system of equations

$$\left\{\begin{array}{c} \frac{d}{dt}C=\alpha_{1}C\left[1-\frac{C}{k_{1}\left(E\right)}-\frac{{\beta^{\prime }}_{1}T}{k_{1}\left(E\right)}\right]\\ \;\;\;-\mu_{1}C-C_{m}\delta_{D}\left(t\right)\\ \frac{d}{dt}E=\alpha_{2}E\left[1-\frac{E}{k_{2}\left(C\right)}\right]-\gamma ET-\mu_{2}E\\ \frac{d}{dt}T=C_{m}\delta_{D}\left(t\right)+\alpha_{3}AT\left(1-\frac{T}{k_{3}}\right)\\ \;\;\;+{\alpha^{\prime }}_{3}T\left[1-\frac{T}{k_{1}\left(E\right)}-\frac{{\beta^{\prime }}_{2}C}{k_{1}\left(E\right)}\right]-\mu_{3}T\\ \frac{d}{dt}P=\gamma ET-\delta P-\mu_{4}P\\ \frac{d}{dt}A=\delta P+\varepsilon TA\left(1-\frac{A}{k_{4}}\right)-\mu_{5}A. \end{array}\right)$$

In this model, an amount *C*_*m *_of normal cells are mutated to cancer cells at time *t *= 0, which is described by the Dirac delta function *δ*_*D *_(*t*): it assumes ∞ at *t *= 0, and 0, otherwise.

Let us simplify the model assuming that *k*_1 _and *k*_2 _are constant, depending only on the status corresponding to cancer free. Under this simplification, we can define the interaction parameters as $\beta_{1}=\beta_{1}^{\prime }\alpha_{1}/k_{1}$ and $\beta_{2}=\beta_{2}^{\prime }\alpha_{3}^{\prime }/k_{1}$. Another simplification assumes that the rate at which cancer cells increase due to new vascularization is much higher than due to existing one, or we let $\alpha_{3}AT\left(1-T/k_{3}\right)\gg \alpha_{3}^{\prime }T\left(1-T/k_{1}\right)$. The above impulsive system taking into account these simplifications can be written as, for *t *> 0,

(1) $$\left\{\begin{array}{c} \frac{d}{dt}C=\alpha_{1}C\left(1-\frac{C}{k_{1}}\right)-\beta_{1}CT-\mu_{1}C\\ \frac{d}{dt}E=\alpha_{2}E\left(1-\frac{E}{k_{2}}\right)-\gamma ET-\mu_{2}E\\ \frac{d}{dt}T=\alpha_{3}AT\left(1-\frac{T}{k_{3}}\right)-\beta_{2}CT-\mu_{3}T\\ \frac{d}{dt}P=\gamma ET-\delta P-\mu_{4}P\\ \frac{d}{dt}A=\delta P+\varepsilon TA\left(1-\frac{A}{k_{4}}\right)-\mu_{5}A, \end{array}\right)$$

in order to describe a perturbation introduced at *t *= 0 in a cancer free status. Hence, the initial conditions supplied to the system of equations become:

(2) $$\begin{array}{l} (C(0)=C_{0}-C_{m},E(0)=E_{0},\\ T(0)=C_{m},P(0)=0,A(0)=0), \end{array}$$

where *C*_0 _and *E*_0 _are the cancer free steady state values (see below), and *C*_*m *_is the instantaneous mutation of normal cells to initiate cancer growth. The vital dynamics of normal cells is similar than that presented in [11]. In Table 1 we present the summary of variables, which depend on time, for instance *C*(*t*), and parameters, which do not depend on time, of the model.

**Table 1:** Symbols and definitions

Symbols	Definitions
*C*	Concentration of normal cells at time t
*E*	Concentration of epithelial cells at time t
*T*	Concentration of cancer cells at time t
*P*	Concentration of pre-angiogenesis cells at time t
*A*	Concentration of angiogenesis cells at time t
*α*_1_	Intrinsic growth rate of normal cells
*α*_2_	Intrinsic growth rate of epithelial cells
*α*_3_	Intrinsic growth rate of cancer cells
*ε*	Intrinsic growth rate of angiogenesis cells
*μ*_1_	Mortality rate of normal cells
*μ*_2_	Mortality rate of epithelial cells
*μ*_3_	Mortality rate of cancer cells
*μ*_4_	Mortality rate of pre-angiogenesis cells
*μ*_5_	Mortality rate of angiogenesis cells
*k*_1_	Carrying capacity of normal cells
*k*_2_	Carrying capacity of epithelial cells
*k*_3_	Carrying capacity of cancer cells
*k*_4_	Carrying capacity of angiogenesis cells
*δ*	Transfer rate from pre-angiogenesis to angiogenesis cells
*γ*	Epithelial sprouting rate
*β*_1_	Rate of inhibition of normal cells by cancer cells
*β*_2_	Rate of inhibition of cancer cells by normal cells

The summary of the variables and parameters of the model.

We next obtain the steady states of the system of equations (1), and the corresponding characteristic equations in order to establish the stability of the steady states.

### Steady states

We determine the steady states of the system (1), denoted by $\bar{Q}=\left(\bar{C},Ē,\bar{T},\bar{P},Ā\right)$. The coordinates of the equilibrium point are obtained by letting the time derivatives in equation (1) equal to zero, for instance, $\frac{d}{dt}C=0$. The first is the equilibrium where there is not any cells, that is,

$${\bar{Q}}^{abs}=\left(0,0,0,0,0\right).$$

For obvious reason, this point is not considered here.

The trivial equilibrium point, given by

$${\bar{Q}}^{0}=\left(C_{0},E_{0},0,0,0\right),$$

where *C*_0 _and *E*_0 _are

(3) $$\left\{\begin{array}{c} C_{0}=\frac{k_{1}}{\alpha_{1}}\left(\alpha_{1}-\mu_{1}\right)\\ E_{0}=\frac{k_{2}}{\alpha_{2}}\left(\alpha_{2}-\mu_{2}\right), \end{array}\right)$$

corresponds to the absence of cancer. The trivial equilibrium point exists for *α*_1 _>*μ*_1 _and *α*_2 _>*μ*_2_. In this model the normal and epithelial cells are not dependent on each other. One possibility of dependence can be done by the carrying capacities *k*_1_(*E*_0_) and *k*_2_(*C*_0_).

The coordinates of the non-trivial equilibrium point ${\bar{Q}}^{*}$ are

(4) $$\left\{\begin{array}{c} \bar{C}=\frac{k_{1}}{\alpha_{1}}\left[\left(\alpha_{1}-\mu_{1}\right)-\beta_{1}\bar{T}\right]\\ Ē=\frac{k_{2}}{\alpha_{2}}\left[\left(\alpha_{2}-\mu_{2}\right)-\gamma \bar{T}\right]\\ \bar{P}=\frac{\gamma k_{2}}{\left(\mu_{4}+\delta \right)\alpha_{2}}\left[\left(\alpha_{2}-\mu_{2}\right)-\gamma \bar{T}\right]\bar{T}\\ Ā=\frac{k_{3}}{k_{3}-\bar{T}}\left[\frac{\mu_{3}}{\alpha_{3}}+\frac{\beta_{2}k_{1}}{\alpha_{3}\alpha_{1}}\left(\alpha_{1}-\mu_{1}\right)-\frac{\beta_{1}\beta_{2}k_{1}}{\alpha_{3}\alpha_{1}}\bar{T}\right], \end{array}\right)$$

where $\bar{T}$ is the positive solution of the equation

(5) $$f\left(T\right)=g\left(T\right)\equiv g_{1}\left(T\right)+g_{2}\left(T\right),$$

with the fourth degree polynomial *f*(*T*), and the third degree polynomials *g*_1_(*T*) and *g*_2_(*T*) being given by

(6) $$\left\{\begin{array}{c} f\left(T\right)=\frac{\gamma \delta k_{2}}{\left(\mu_{4}+\delta \right)\mu_{5}\alpha_{2}k_{3}}\left[\left(\alpha_{2}-\mu_{2}\right)-\gamma T\right]\\ \;\;\;\times {\left(k_{3}-T\right)}^{2}T\\ g_{1}\left(T\right)=\left(1-\frac{\varepsilon }{\mu_{5}}T\right)\left(k_{3}-T\right)\\ \;\;\;\times \left[\frac{\mu_{3}}{\alpha_{3}}+\frac{\beta_{2}k_{1}}{\alpha_{3}\alpha_{1}}\left(\alpha_{1}-\mu_{1}\right)-\frac{\beta_{1}\beta_{2}k_{1}}{\alpha_{3}\alpha_{1}}T\right]\\ g_{2}\left(T\right)=\frac{\varepsilon k_{3}}{\mu_{5}k_{4}}T\\ \;\;\;\times {\left[\frac{\mu_{3}}{\alpha_{3}}+\frac{\beta_{2}k_{1}}{\alpha_{3}\alpha_{1}}\left(\alpha_{1}-\mu_{1}\right)-\frac{\beta_{1}\beta_{2}k_{1}}{\alpha_{3}\alpha_{1}}T\right]}^{2}. \end{array}\right)$$

By inspecting $\bar{C}$, *Ē *and *Ā*, the positive solution $\bar{T}$, in order to ${\bar{Q}}^{*}$ be biologically feasible, must satisfy, respectively, the constraints $\bar{T}<T_{C},\bar{T}<T_{E}$ and $\bar{T}<T_{A}$, where

(7) $$\left\{\begin{array}{c} T_{C}=\frac{\alpha_{1}-\mu_{1}}{\beta_{1}}\\ T_{E}=\frac{\alpha_{2}-\mu_{2}}{\gamma }\\ T_{A}=k_{3}. \end{array}\right)$$

However, the constraint for the equilibrium *Ā *is $\bar{T}<T_{g}$, where

(8) $$\begin{array}{ll} T_{g} & =\frac{\alpha_{1}\alpha_{3}}{\beta_{1}\beta_{2}k_{1}}\left[\frac{\mu_{3}}{\alpha_{3}}+\frac{\beta_{2}k_{1}}{\alpha_{3}\alpha_{1}}\left(\alpha_{1}-\mu_{1}\right)\right]\;\\ & =\frac{\alpha_{1}\mu_{3}}{\beta_{1}\beta_{2}k_{1}}+\frac{\alpha_{1}-\mu_{1}}{\beta_{1}},\;\\ & \; \end{array}$$

but it is always satisfied when $\bar{T}<T_{C}$, since *T*_*g *_>*T*_*C*_.

With respect to the constraints, we must have $\bar{T}<\text{min}\left\{T_{C},T_{E},T_{A}\right\}$, where min{*T*_*C*_, *T*_*E*_, *T*_*A*_} is the minimum among *T*_*C*_, *T*_*E *_and *T*_*A*_. The constraints depend inversely with removing parameters *β*_1 _and *γ*, and directly with the carrying capacity *k*_3_. Hence, for higher values of removing parameters *β*_1 _and *γ*, and lower values of carrying capacity *k*_3_, the constraints are decreased, that is, the steady state cancer cells $\bar{T}$ must assume lower values. Let us suppose that *k*_3 _is high, which implies that cancer cells $\bar{T}$ can achieve higher values. If the sproutings rate *γ *from existing blood vessels is high, in principle it seems to be beneficial to cancer cells. However, higher values of *γ *result in lower values for the constraint *T*_*E*_, and, consequently, $\bar{T}$ must assume lower values, because it must be lower than both *T*_*E *_and *k*_3_. Therefore, cancer cells will achieve higher values for high carrying capacity *k*_3_, and lower sprouting rate *γ*. Further angiogenesis cells must be originated by the clonal division from a small amount of pre-angiogenesis cells. In appendix A we show the analysis of equation (5). Summarizing, the number of solutions $\bar{T}$ depends on the values assigned to the model's parameters, which can be 0, 2 or 4. Notwithstanding, the solution $\bar{T}$ must obey the constraints given in equation (7), which reduces the number of solutions up to 2. In the case of 2 solutions, we denote the solutions $\bar{T}$ as small (*T*_*<*_, and the corresponding equilibrium point ${\bar{Q}}_{<}^{*}$) and big (*T*_>_, and the corresponding equilibrium point ${\bar{Q}}_{>}^{*}$). Hence, the constraints play an important role in the number of equilibrium points. Notice that the interacting parameter *ε *does not appear in the constraints. Let us analyze possible scenarios resulting by varying the non-linear term parameters *β*_1_, *β*_2_, *γ *and *ε*. First, let us vary *β*_1_. Notice that *T*_*C *_depends on *β*_1_, but *T*_*E *_and *T*_*A *_do not. For small *β*_1_, the solution $\bar{T}$ of equation (5) situates below *T*_*C*_. However, as *β*_1 _increases, $\bar{T}$ can surpass *T*_*C*_, after intercepting *T*_*C *_at $\beta_{1}=\beta_{1}^{c}$. As $\bar{T}$ increases with *β*_1_, $\bar{C}$, given by the first equation of (4), decreases and reaches zero at $\beta_{1}=\beta_{1}^{c}$, and $\bar{C}<0$ sinceafter. At $\beta_{1}=\beta_{1}^{c}$ we have $\bar{T}=T_{C}^{c}=\left(\alpha_{1}-\mu_{1}\right)/\beta_{1}^{c}$. Let us suppose that $T_{C}^{c}<\text{min}\left\{T_{E},T_{A}\right\}$, the minimum between *T*_*E *_and *T*_*A*_. When $\bar{C}=0$, there arises another feasible equilibrium point ${\bar{Q}}^{c}$, with the coordinates

(9) $$\left\{\begin{array}{c} \bar{C}=0\\ Ē=\frac{k_{2}}{\alpha_{2}}\left[\left(\alpha_{2}-\mu_{2}\right)-\gamma \bar{T}\right]\\ \bar{P}=\frac{\gamma k_{2}}{\left(\mu_{4}+\delta \right)\alpha_{2}}\left[\left(\alpha_{2}-\mu_{2}\right)-\gamma \bar{T}\right]\bar{T}\\ Ā=\frac{k_{3}\mu_{3}}{\alpha_{3}\left(k_{3}-\bar{T}\right)}, \end{array}\right)$$

where $\bar{T}=T_{C}^{c}$, and the equilibrium $\bar{T}$ does not depend anymore on the interacting parameter *β*_1_. Hence for $\beta_{1}\geq \beta_{1}^{c}$ we have a constant value $\bar{T}=\left(\alpha_{1}-\mu_{1}\right)/\beta_{1}^{c}$, which implies in constant values for *Ē*, $\bar{P}$ and *Ā*.

See more discussion below, in the stability analysis.

The equilibrium point ${\bar{Q}}^{c}$ corresponds to the case where the cancer cells displaced normal cells. By inspecting the equation for $\bar{C}$, the first in equation (4), the normal cells drop out to zero for a sufficiently higher values of *β*_1_. In another words, when cancer cells have very higher fitness than normal cells and overcome the competition for resources, they can lead to the exclusion of the normal cells (of course, the cancer diseased person dies before reaching this equilibrium). Notice that ${\bar{Q}}^{c}$ can be avoided as the equilibrium point when *k*_3 _assumes small values, that is, if *k*_3 _< min{*T*_*E*_, *T*_*C*_}, minimum between *T*_*E *_and *T*_*C*_. In this situation, the equilibrium value $\bar{T}$ is always smaller than *k*_3_, and all coordinates of the equilibrium point are positive (Appendix A).

Second, let us now vary *β*_2_. The third equation of (1) in the equilibrium can be written as

$$\bar{T}=\frac{k_{3}}{Ā\alpha_{3}}\left[\left(Ā\alpha_{3}-\mu_{3}\right)-\beta_{2}\bar{C}\right].$$

When *β*_2 _increases, $\bar{T}$ decreases, and at a certain value of *β*_2_, say $\beta_{2}^{th},\bar{T}$ assumes zero value. When $\bar{T}=0$, the unique feasible equilibrium point is the trivial ${\bar{Q}}_{0}$. When the influence of the normal cells and the environment is higher ($\beta_{2}\geq \beta_{2}^{th}$), then cancer can not be maintained.

Cancer onset depends on the interplay among the initial condition *T*(0) = *C*_*m *_and interacting parameters *β*_1 _and *β*_2_. Once cancer cells are originated by mutation from several sources, these cells interact with healthy cells and the surrounding environment. While the parameter *β*_1 _is practically insensitive in the behavior of the dynamics, however, there is a threshold in the parameter *β*_2_, above which cancer can not be established. This result shows that cancer is rarely induced in organs (or tissues) displaying an efficient (numerically and functionally) reparative or regenerative mechanism [12], which is the reason for the incidence of cancer rising exponentially with age [13].

Finally, let us analyze the effects of varying the sprouting rate *γ *and angiogenesis increasing rate *ε*.

When *γ *increases, *Ē *decreases according to the second equation of (4). Let us assume that *Ē *reaches zero at a certain value named *γ*^*c*^, and *Ē *< 0 sinceafter. When *Ē *= 0, differently from $\bar{C}=0$, the unique feasible equilibrium point is the trivial ${\bar{Q}}_{0}$. Hence, we expect that *Ē *> 0 for *γ *≥ 0, because of $\bar{T}<T_{E}=\left(\alpha_{2}-\mu_{2}\right)/\gamma$ is always obeyed, and there is not a critical value *γ*^*c*^. When we decrease *γ*, we observe that equation (5) may not have biologically feasible solutions.

Initially, let us analyze *γ *= 0. In this case, we have *f*(*T*) = 0, and the equilibrium value $\bar{T}$ is solution of the polynomial of degree two

(10) $$\begin{array}{ll} 0 & =\left(1-\frac{\varepsilon }{\mu_{5}}T\right)\left(k_{3}-T\right)+\frac{\varepsilon k_{3}}{\mu_{5}k_{4}}T\;\\ & \;\times \left[\frac{\mu_{3}}{\alpha_{3}}+\frac{\beta_{2}k_{1}}{\alpha_{3}\alpha_{1}}\left(\alpha_{1}-\mu_{1}\right)-\frac{\beta_{1}\beta_{2}k_{1}}{\alpha_{3}\alpha_{1}}T\right],\;\\ & \; \end{array}$$

with another being given by $\bar{T}=T_{g}$. Moreover, from equation (4), we have $\bar{P}=0$ (due to *γ *= 0, the role of the parameter *δ *does not matter). In this case, there arises another feasible equilibrium point ${\bar{Q}}^{p}$, with the coordinates

(11) $$\left\{\begin{array}{c} \bar{C}=\frac{k_{1}}{\alpha_{1}}\left[\left(\alpha_{1}-\mu_{1}\right)-\beta_{1}\bar{T}\right]\\ Ē=\frac{k_{2}}{\alpha_{2}}\left(\alpha_{2}-\mu_{2}\right)\\ \bar{P}=0\\ Ā=\frac{k_{3}}{k_{3}-\bar{T}}\left[\frac{\mu_{3}}{\alpha_{3}}+\frac{\beta_{2}k_{1}}{\alpha_{3}\alpha_{1}}\left(\alpha_{1}-\mu_{1}\right)-\frac{\beta_{1}\beta_{2}k_{1}}{\alpha_{3}\alpha_{1}}\bar{T}\right]. \end{array}\right).$$

Equation (10) has two positive solutions (not shown) when *T*_*g *_>*k*_3 _and *ε > μ*_5_/*k*_3_, where *T*_*g *_is given by equation (8), while a unique positive solution occurs when *T*_*g *_*= k*_3_. Let us introduce a threshold of *ε*, named *ε*^*th*^. We remark that this threshold value appears only for *γ *= 0. Hence, when *ε *>*ε*^*th*^, with *ε*^*th *^>*μ*_5_/*k*_3_, we have two positive solutions, named $T_{<}^{\varepsilon }$ (and the corresponding equilibrium point ${\bar{Q}}_{<}^{p}$) and $T_{>}^{\varepsilon }$ (and the corresponding equilibrium point ${\bar{Q}}_{>}^{p}$), which collapse to one at *ε *= *ε*^*th*^, and for *ε *<*ε*^*th *^there is not positive solution. The equilibrium point ${\bar{Q}}^{p}$ represents the origin of angiogenesis from other sources, not from existing vascular system feeding an organ of the human body. Two facts must occur to appearing of ${\bar{Q}}^{p}$: sufficiently higher capacity of cancer cells to originate new vascular system from external sources (*ε *>*ε*^*th*^), and higher initial amount, at time 0, of vascular cells $\left(T\left(0\right)>T_{<}^{\varepsilon }\right)$.

Now, for lower values of *γ *(*γ *≳ 0), as a consequence of the appearing of *ε*^*th *^for *γ *= 0, equation (5) does not have positive solution and there is not biologically feasible equilibrium point. Let us introduce a threshold of *γ*, named *γ*^*th*^. For *γ *<*γ*^*th *^the unique solution is $\bar{T}=0$ (this fact is shown numerically). Note that as *ε *increases, *γ*^*th *^decreases. Hence, higher the capacity of building up new vascularization *ε*, less the amount of sprouts originated from existing blood vessels needed to angiogenesis. In the special case *ε *= 0 (cancer cells do not promote growth in the new vascularization), the equilibrium $\bar{T}$, which must be substituted in the variables given in equation (4), must be solution of *f*(*T*) = *g*(*T*), where

(12) $$\left\{\begin{array}{c} \begin{array}{c} f\left(T\right)=\frac{\gamma \delta k_{2}}{\left(\mu_{4}+\delta \right)\mu_{5}\alpha_{2}k_{3}}\left[\left(\alpha_{2}-\mu_{2}\right)-\gamma T\right]\\ \;\;\;\;\times \left(k_{3}-T\right)T \end{array}\\ g\left(T\right)=\frac{\mu_{3}}{\alpha_{3}}+\frac{\beta_{2}k_{1}}{\alpha_{3}\alpha_{1}}\left(\alpha_{1}-\mu_{1}\right)-\frac{\beta_{1}\beta_{2}k_{1}}{\alpha_{3}\alpha_{1}}T. \end{array}\right)$$

This equation clearly shows that a positive solution $\bar{T}$ is feasible for sufficiently higher values of *γ*, that is, $\gamma >\gamma_{0}^{th}$, where $\gamma_{0}^{th}$ corresponds to *ε *= 0. Due to the fact that *ε *increases as *γ*^*th *^decreases, $\gamma_{0}^{th}$ is the upper bound of the threshold of *γ*. Even the cancer cells do not promote the proliferation of the sprouted endothelial (pre-angiogenesis) cells (*ε *= 0), if the capacity of endothelial cell sprouting is higher (*γ *>*γ*^*th*^), then cancer cells can be maintained.

There are two mechanisms by which the tumor's vasculature is built: (1) as a tumor grows, it excretes tumor angiogenesis factor, which help activate endothelial cells of nearby blood vessels, initiating angiogenesis; (2) bone marrow derived endothelial progenitor cells are mobilized into the blood, by means of long-range signaling, and they are recruited from blood by the tumor by short-range signaling, resulting in vasculogenesis (the vascular endothelium of the nearby vessels become activated and allows the endothelial progenitor cells to extravase and start a cycle of differentiation/division) [14]. In both cases, the newly stimulated endothelial cells (described by parameter *γ*) and the recruited endothelial progenitor cells (this phenomenon is not considered in the model; however, the case *γ *= 0 can in some extent be understood as vasculogenesis) enter a stage of clonal expansion and continue to form blood vessels, which is described by the parameter *ε*.

With respect to the linear term of the pre-angiogenesis parameter *δ*, when $\delta \to \infty ,{\bar{Q}}^{\infty }$ appears, similar to the equilibrium point ${\bar{Q}}^{p}$, because $\underset{\delta \to \infty }{\text{lim}}\bar{P}=0$. However, from the third equation of (4), we have $\underset{\delta \to \infty }{\text{lim}}\delta \bar{P}=\gamma Ē\bar{T}$. Hence, the coordinates of ${\bar{Q}}^{\infty }$ are those given in equation (11), but $\bar{T}$ is solution of the equation *f*(*T*) = *g*_1_(*T*) + *g*_2_(*T*), where

$$f\left(T\right)=\frac{\gamma k_{2}}{\mu_{5}\alpha_{2}k_{3}}\left[\left(\alpha_{2}-\mu_{2}\right)-\gamma T\right]{\left(k_{3}-T\right)}^{2}T,$$

and *g*_1_(*T*) + *g*_2_(*T*) is given be equation (6). Notice that *f*(*T*) differs from that in equation (6) by the factor *δ*/(*μ*_4 _+ *δ*). The equilibrium point ${\bar{Q}}^{\infty }$ represents the absence of the intermediate pre-angiogenesis, that is, angiogenesis occurs directly and instantaneously from epithelial cells.

Summarizing, the non-trivial equilibrium points ${\bar{Q}}^{*}$ (${\bar{Q}}_{<}^{*}$ and ${\bar{Q}}_{>}^{*}$, when they exist), ${\bar{Q}}^{c}$ (for $\beta_{1}\geq \beta_{1}^{c}$), and ${\bar{Q}}^{p}$ (when *γ *= 0) are biologically feasible, but ${\bar{Q}}^{c}$ is not a real cancer equilibrium point, since $\bar{C}=0$. Especially, ${\bar{Q}}^{p}$ is a cancer equilibrium point only if vasculogenesis can act alone to generate new blood vessels. The analysis of the model showed the existence of thresholds $\beta_{2}^{th}$ and *γ*^*th *^(and, eventually, *ε*^*th*^, for *γ *= 0), and a critical value $\beta_{1}^{c}$.

We analyzed the steady states of the model described by the system of ordinary differential equations (1) assuming that all parameters of the linear terms (*μ*_1_*, μ*_2_, *μ*_3_, *μ*_4_, *μ*_5 _and *δ*) plus the intrinsic growth rates *α*_1_, *α*_2 _and *α*_3 _and carrying capacities *k*_1_, *k*_2_, *k*_3 _and *k*_4 _are fixed values. But, the parameters regarded to the interaction between different cells (*β*_1_, *β*_2_, *γ *and *ε*) are generally unknown, hence they were allowed to vary to analyze broad range of variation in these parameters.

### Local stability analysis

The local stability of the equilibrium points [15] is determined by the eigenvalues of the characteristic equation det $\left(\bar{J}-\lambda I\right)=0$, where $\bar{J}$ is the Jacobian *J *evaluated at the equilibrium point under analysis,

$$\bar{J}=\left[\begin{array}{ccccc} -\frac{\alpha_{1}}{k_{1}}\bar{C} & 0 & -\beta_{1}\bar{C} & 0 & 0\\ 0 & -\frac{\alpha_{2}}{k_{2}}Ē & -\gamma Ē & 0 & 0\\ -\beta_{2}\bar{T} & 0 & -\frac{\alpha_{3}}{k_{3}}Ā\bar{T} & 0 & j_{1}\\ 0 & \gamma \bar{T} & \gamma Ē & -j_{2} & 0\\ 0 & 0 & j_{3} & \delta & -j_{4} \end{array}\right]$$

where *j*_1_, *j*_2_, *j*_3 _and *j*_4 _are

(13) $$\left\{\begin{array}{c} j_{1}=\alpha_{3}\bar{T}\left(1-\frac{\bar{T}}{k_{3}}\right)\\ j_{2}=\mu_{4}+\delta \\ j_{3}=\varepsilon Ā\left(1-\frac{Ā}{k_{4}}\right)\\ j_{4}=\delta \frac{\bar{P}}{Ā}+\frac{\varepsilon }{k_{4}}Ā\bar{T}. \end{array}\right)$$

The Jacobian $\bar{J}$ was obtained using equilibrium equations given in (4) assuming that $\bar{C}\neq 0$. When $\bar{C}=0$, the element in the first line and first column is $a_{11}=\left(\alpha_{1}-\mu_{1}\right)-\beta_{1}\bar{T}$, and in the third column, *a*_13 _= 0.

We present analytical results corresponding to the equilibrium points ${\bar{Q}}^{0}$ and ${\bar{Q}}^{c}$.

The eigenvalues (from Jacobian ${\bar{J}}^{0}$) corresponding to the trivial equilibrium point ${\bar{Q}}^{0}$ are: *λ*_1 _= -*μ*_5_, *λ*_2 _= - (*μ*_4 _+ *δ), λ*_3 _= - (*μ*_3 _+ *β*_2_*C*_0_), *λ*_4 _= - (*α*_1 _- *μ*_1_), and *λ*_5 _= - (*α*_2 _- *μ*_2_). In the last two eigenvalues we used the coordinates given by equation (3). Hence, the trivial equilibrium ${\bar{Q}}^{0}$ is locally asymptotically stable whenever this point exists (*α*_1 _>*μ*_1 _and *α*_2 _>*μ*_2_), because all eigenvalues become negative.

The non-trivial equilibrium point ${\bar{Q}}^{c}$, with coordinates given in equation (9), has one of them zero ($\bar{C}=0$). The eigenvalues corresponding to the trivial equilibrium point ${\bar{Q}}^{c}$ are obtained from the Jacobian ${\bar{J}}^{c}$. Once $\bar{C}=0$, one of the eigenvalues is $\lambda_{1}=-\beta_{1}\left(\bar{T}-T_{C}\right)$, arising one of the conditions to the not real cancer equilibrium ${\bar{Q}}^{c}$ being stable, that is, $\bar{T}>T_{C}$, where *T*_*C *_is given by equation (7). The remaining four eigenvalues are obtained from the sub-matrix ${\bar{J}}_{1}^{c}$, which comes from ${\bar{J}}^{c}$ excluding first row and first column. Let us define matrix *A *as $A=-{\bar{J}}_{1}^{c}$, or,

(14) $$A=\left[\begin{array}{cccc} \frac{\alpha_{2}}{k_{2}}Ē & \gamma Ē & 0 & 0\\ 0 & \frac{\alpha_{3}}{k_{3}}Ā\bar{T} & 0 & -j_{1}\\ -\gamma \bar{T} & -\gamma Ē & j_{2} & 0\\ 0 & -j_{3} & -\delta & j_{4} \end{array}\right],$$

where *j*_1_, *j*_2_, *j*_3 _and *j*_4 _were defined in equation (13). In appendix B we show that, for big solution *T*_>_, *A *is an *M*-matrix, and, hence, all eigenvalues associated to ${\bar{J}}_{1}^{c}$ have negative real part. Hence, ${\bar{Q}}^{c}$, which is biologically feasible but not real cancer, is locally asymptotically stable only if $\bar{T}>T_{C}$. Therefore, ${\bar{Q}}^{c}$ appears when one of the constraints to ${\bar{Q}}^{*}$ be biologically feasible is violated: in the equation (4), instead of $\bar{C}<0$, we have $\bar{C}=0$ for $\beta_{1}\geq \beta_{1}^{c}$.

The local stability of the non-trivial equilibrium ${\bar{Q}}^{*}$ is performed numerically. However, we stress the fact that the equilibrium ${\bar{Q}}^{c}$ is an extension of ${\bar{Q}}^{*}$ for $\beta_{1}\geq \beta_{1}^{c}$. Hence, the big solution *T*_>_of equation (5) for $\beta_{1}\geq \beta_{1}^{c}$ must be stable. For this reason we showed the local stability of ${\bar{Q}}^{c}$, even though this is not a real cancer equilibrium.

## Results and Discussion

In this section we present numerical simulations of the model, which are discussed. The numerical methods used are bisection (to find zeros of polynomials) and 4^*th *^order Runge-Kutta (to solve system of ordinary differential equations) [16].

### Numerical analysis of the model

Numerical simulations are performed taking into account the values and units of the model's parameters given in Table 2. These values have the purpose of illustrating the outcomes of the model. Cancer free equilibrium point ${\bar{Q}}^{0}$ has the cancer free concentrations: *C*_0 _= 9 *cells/uv *and *E*_0 _= 10 *cells/uv*, where *uv *stands for an arbitrarily unit of volume. Hereafter we will omit units of all variables and parameters. In this section we deal with the steady state of the system (1), determining the equilibrium points and bifurcation diagrams. We also study the dynamical trajectories of the system (1) assuming that mutations occurred and cancer cells arise suddenly. For this reason the initial conditions are given by equation (2). The reason behind this is the fact that the trivial equilibrium point ${\bar{Q}}^{0}$ always exists and is stable. Hence, the initial conditions supplied to the system of equations correspond to an appearance of *C*_*m *_number of cancer cells in a cancer free situation ${\bar{Q}}^{0}$, that is, at time *t *= 0 the variables assume *C*(0) = *C*_0 _- *C*_*m*_, *E*(0) = *E*_0_, *T*(0) = *C*_*m*_*, P*(0) = 0 and *A*(0) = 0. The initial amount of cancer cells is originated by mutation from several sources. In some special situations we will use *C*(0) = *C*_0_. The initial amount of mutations *C*_*m *_can either grow up to cancer, or fades out.

**Table 2:** Values of the parameters

Parameters	Fixed values	Alternative values**	Units
α_1_	0.1		*days*^-1^
α_2_	0.1		*days*^-1^
α_3_	0.2	5.0	[*A*]^-1 ^× *days*^**-1**^
*ε*	0.01*	0.1	[*T*]^-1 ^× *days*^-1^
*μ*_1_	0.01		*days*^-1^
*μ*_2_	0.05		*days*^-1^
*μ*_3_	0.05	0.005	*days*^-1^
*μ*_4_	0.01		*days*^-1^
*μ*_5_	0.01		*days*^-1^
*k*_1_	10		[*C*]
*k*_2_	20		[*E*]
*k*_3_	5	0.1	[*T*]
*k*_4_	1	0.2	[*A*]
*δ*	0.1		*days*^-1^
*γ*	0.01*	0.02	[*T*]^-1 ^× *days*^-1^
*β*_1_	0.01*		[*T*]^-1 ^× *days*^-1^
*β*_2_	0.01*		[*C*]^-1 ^× *days*^-1^

The values for model's parameters. The unity of [•] is number of cells of type • per unit of volume, •*/uv*. Parameters regarded to the non-linear terms of the dynamical system are allowed to vary (indicated by the symbol*). The values of the parameters that differ from the fixed values are in the column indicated with **.

Based on the values given in Table 2, the constraints, using equations (7) and (8), are *T*_*C *_= 9.0 (at *β*_1 _= 0.01), *T*_*E *_= 5.0, *T*_*A *_= 5.0 and *T*_*g *_= 14.0. The coordinates of the non-trivial equilibrium points, corresponding to small (*T*_*<*_) and big (*T*_*>*_) equilibrium values of cancer cells obtained from equation (5), are, respectively, ${\bar{Q}}_{<}^{*}=\left(8.92,9.85,0.08,0.07,0.71\right)$ and ${\bar{Q}}_{>}^{*}=\left(5.32,2.64,3.68,0.88,1.96\right)$, from equation (4). The corresponding five eigenvalues (*λ*_*i*_, *i *= 1, ..., 5) are given in Table 3, showing that the small is unstable (*λ*_1 _> 0), while the big is stable (all *λ*_*i*_'s are negative). The big equilibrium *T*_*>*_= 3.68 satisfies the constraints given in equation (7). The trivial equilibrium ${\bar{Q}}^{0}$ is always stable.

**Table 3:** Eigenvalues

Eigenvalues	**Corresponding to **$Q_{<}^{*}$	**Corresponding to **$Q_{>}^{*}$
*λ*_1_	+0.028	-0.227 - *i*0.024
*λ*_2_	-0.089 - *i*0.060	-0.227+ *i*0.024
*λ*_3_	-0.089 + *i*0.060	-0.068
*λ*_4_	-0.064	-0.0293 - *i*0.0059
*λ*_5_	-0.047	-0.0293 + *i*0.0059

Eigenvalues (*λ*_*i*_, *i *= 1,..., 5) corresponding to the non-trivial equilibrium points ${\bar{Q}}_{<}^{*}$ (unstable) and ${\bar{Q}}_{>}^{*}$ (stable) using values given in Table 2.

**Figure 1:**
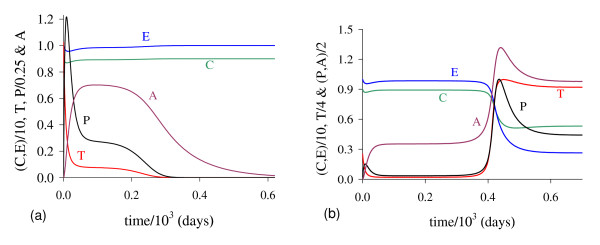
**Dynamical trajectories using values in Table 2**. Dynamical trajectories of the system (1) considering the values of parameters given in Table 2. The initial conditions determine the region of attraction: trivial ${\bar{Q}}^{0}$ for *T*(0) = 1.023 (a), or non-trivial ${\bar{Q}}_{>}^{*}$ (b). The scales of vertical and horizontal axes must be multiplied by the factors shown in the legends to obtain the actual values.

In Figure 1 we illustrate the dynamical trajectories of system (1) taking into account the values of parameters given in Table 2. The dynamical trajectories depend on the initial conditions. In Figure 1(a), due to *T*(0) = 1.023, the dynamical trajectories are attracted to the trivial equilibrium ${\bar{Q}}^{0}$; while for *T*(0) = 1.024, the dynamical trajectories attain the non-trivial equilibrium ${\bar{Q}}_{>}^{*}$ (Figure 1(b), which shows a sudden relapse to cancer onset at around 400 *days*). The exact critical amount of cancer cells T* that divides two regions of attraction situates in the open interval (1.023,1.024). Notice that T* is much higher than *T*_*<*_= 0.08, which comes out due to other initial conditions. However, if the initial conditions are the coordinates of ${\bar{Q}}_{<}^{*}$, except *T*(0), that is, *C*(0) = 8.92, *E*(*0*) = 9.85, *P*(0) = 0.07 and *A*(*0*) = 0.71, then, for an arbitrary value *ϵ *> 0, the dynamical trajectories go to trivial ${\bar{Q}}^{0}$ when *T*(0) = (1 - *ϵ*) × 0.08, and to the non-trivial ${\bar{Q}}_{>}^{*}$ when *T*(0) = (1 + *ϵ*) × 0.08 (for instance, *ϵ *= 0.001, figures not shown). In this case, we have T* = *T*_*<*_. Hence, ${\bar{Q}}_{<}^{*}$ is the break-point, and its coordinates generate a hyper-surface that divides two attracting regions (see Appendix A). Notice that *T*(0) = 1.023 which plays the role of separating two attracting regions is 12-fold higher than *T*_<_= 0.08.

The initial amount of normal cells that suffer mutation *C*_*m *_is crucial to trigger cancer disease. Figure 1 shows that if this amount is below a critical value, that is, *T*(0) = *C*_*m *_<T*, then repairing systems act efficiently and cancer does not settle in an organ. However, if the mutated cells surpass the critical value, the repairing systems do not avoid the onset of cancer.

Next, numerical simulations are performed in order to assess the effects of varying parameters *β*_1_, *β*_2_, *γ *and *ε*. In all dynamical trajectories, remember that unstable solution *T*_*<*_is very small, but *T*(0) is higher due to the initial conditions supplied to system (1) correspond to the coordinates of the trivial equilibrium ${\bar{Q}}^{0}$, and not those of the unstable ${\bar{Q}}_{<}^{*}$. Hence, *T*(0) is not comparable with *T*_*<*_, one of the coordinates of the break-point ${\bar{Q}}_{<}^{*}$. The cancer cells proliferate above the subclinical threshold of 10^3 ^cells and reach 10^9 ^cells which is the X-ray detectable threshold [11].

#### Interaction between normal and cancer cells - *β*_1 _and *β*_2_

Direct competition between normal and cancer cells for resources and space occur in order to grow. But there are many factors that affects both populations, like indirect interaction as excretion released by cells, changes in the environment and control mechanisms. The parameters *β*_1 _and *β*_2 _take into account these factors also.

Let us assess the cancer cells affecting negatively in the normal cells, by varying *β*_1_.

**Figure 2:**
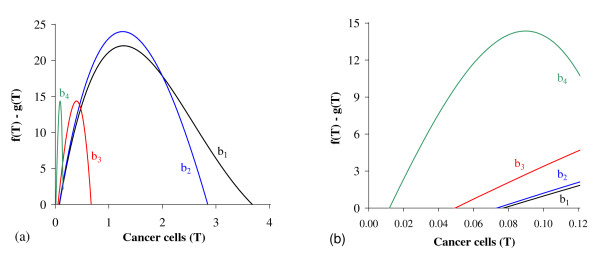
**Two Positive solutions for four values of *β***_**1**_. Two positive roots for the equation *f*(*T*) = *g*(*T*), for four values of *β*_1_. In (a) we show both roots, while in (b), a zoom near lower values of *β*_1_. Legends of curves stand for: *b*_1 _= 0.01 (Table 2), *b*_2 _= 0.1, *b*_3 _= 1.0 and *b*_4 _= 10.0.

In Figure 2 we show $\bar{T}$, the solutions of *f*(*T*) - *g*(*T*) = 0. Figure 2(a), which corresponds to the figure shown in Appendix A with *T*_*E *_= *T*_*A *_= 5.0, shows the existence of two positive solutions: small (denoted by *T*_*<*_) and big (denoted by *T*_>_) for four values of *β*_1_: 0.01 (labelled *b*_1_), 0.1 (*b*_2_), 1.0 (*b*_3_) and 10.0 (*b*_4_). In Figure 2(b) a zoom near lower values of *β*_1 _is shown to enhance the small solution *T*_*<*_.

**Figure 3:**
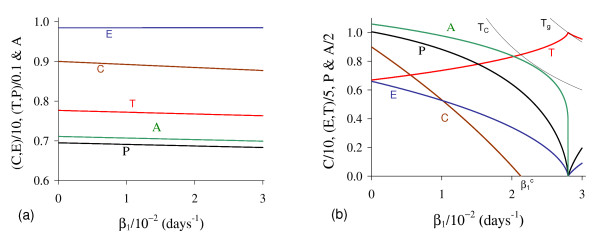
**Positive equilibrium values varying *β***_**1**_. The coordinates of the positive equilibrium points varying *β*_1_. In (a) we show the coordinates of the small equilibrium point ${\bar{Q}}_{<}^{*}$, and in (b), of the big equilibrium point ${\bar{Q}}_{>}^{*}$. The coordinates of ${\bar{Q}}_{<}^{*}$ is quite insensitive with variation in *β*_1_, and in (b) we also show the curves of *T*_*C *_($\bar{C}=0$ at $\bar{T}=T_{C}$) and *T*_*g *_(*Ā *= 0 at $\bar{T}=T_{g}$). The small root *T*_<_decreases very slowly, while the big one *T*_> _increases up to *T*_> _*= T*_*g*_, and then, decreases. The scales of vertical and horizontal axes must be multiplied by the factors shown in the legends to obtain the actual values.

Let us vary *β*_1 _and compute the corresponding equilibrium value $\bar{T}$ using equation (5). As shown in Figure 2, we have two positive solutions: small, *T*_*<*_, and big, *T*_>_. Substituting this solution into equation (4), we obtain the coordinates of the non-trivial equilibrium point ${\bar{Q}}^{*}$. In Figure 3 we show the coordinates of the equilibrium points ${\bar{Q}}_{<}^{*}$ (a) and ${\bar{Q}}_{>}^{*}$ (b). In (b) we also show the curves of *T*_*C *_and *T*_*g*_, which intercept the curve of big solution *T*_>_. When *T*_>_= *T*_*C*_, which occurs at $\beta_{1}=\beta_{1}^{c}=2.1273\times 10^{-2}$, we have $\bar{C}=0$, at which the big non-trivial ${\bar{Q}}_{>}^{*}$ disappears and arises an another equilibrium ${\bar{Q}}_{>}^{c}$, with $\bar{C}=0$ and other coordinates given by equation (9), which has fixed value $T_{>}=T_{C}^{c}=\left(\alpha_{1}-\mu_{1}\right)/\beta_{1}^{c}=4.2306$ for $\beta_{1}\geq \beta_{1}^{c}$. Coordinates of ${\bar{Q}}_{>}^{c}$ are same for all $\beta_{1}\geq \beta_{1}^{c}$. Mathematically, there is another value of *β*_1_, which does not change the existing equilibrium point ${\bar{Q}}^{c}$, at which we have *T*_>_= *T*_*g*_, that is, $\beta_{1}=\beta_{1}^{A}=2.8001\times 10^{-2}$. At this value we have *Ā *= 0, with $T_{>}=T_{g}^{A}=\frac{\alpha_{1}\mu_{3}}{\beta_{1}^{A}\beta_{2}k_{1}}+\frac{\alpha_{1}-\mu_{1}}{\beta_{1}^{A}}=4.9996$.

**Figure 4:**
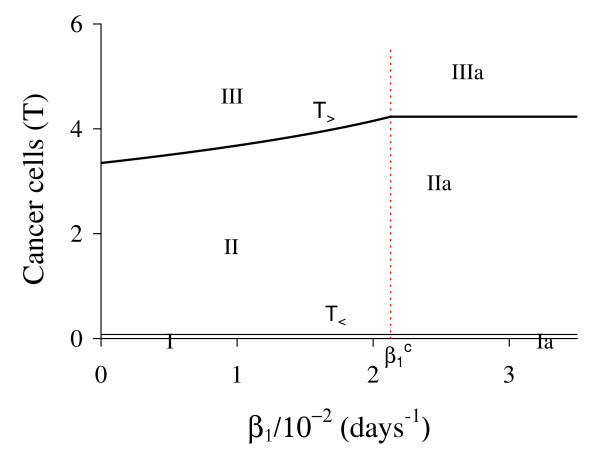
**Bifurcation diagram varying *β***_**1**_. The bifurcation diagram of $\bar{T}$ varying *β*_1_, showing the attracting regions. With respect to the coordinates of small equilibrium point ${\bar{Q}}_{<}^{*}$ for all *β*_1_, the trivial ${\bar{Q}}^{0}$ is attractor for initial conditions in a very small region I (and Ia). Initial conditions in regions II and III are attracted to ${\bar{Q}}_{>}^{*}$ for $\beta_{1}<\beta_{1}^{c}$, and initial conditions in regions IIa and IIIa are attracted to ${\bar{Q}}_{>}^{c}$ for $\beta_{1}\geq \beta_{1}^{c}$. The scales of vertical and horizontal axes must be multiplied by the factors shown in the legends to obtain the actual values.

Figure 3 shows that trivial ${\bar{Q}}^{0}$ and small non-trivial ${\bar{Q}}_{<}^{*}$ exist for all *β*_1_, but big non-trivial is ${\bar{Q}}_{>}^{*}\left(\beta_{1}<\beta_{1}^{c}\right)$ or ${\bar{Q}}_{>}^{c}\left(\beta_{1}\geq \beta_{1}^{c}\right)$. In Figure 4, we show the bifurcation diagram, considering $\bar{T}$ as a function of *β*_1 _(curve *T *in (a) and (b) of Figure 3). For all values of *β*_1 _the small equilibrium point ${\bar{Q}}_{<}^{*}$ is the break-point, which divides two regions where trivial ${\bar{Q}}^{0}$ or big non-trivial ${\bar{Q}}_{>}^{*}$ (or ${\bar{Q}}_{>}^{c}$) is attracting point. Initial conditions set in a small region marked with I and Ia are attracted to the trivial equilibrium point ${\bar{Q}}^{0}$. However, initial conditions set in regions marked with II and III are attracted to big non-trivial equilibrium point ${\bar{Q}}_{>}^{*}$ for $\beta_{1}<\beta_{1}^{c}$, while for $\beta_{1}\geq \beta_{1}^{c}$ (regions marked with IIa and IIIa), to ${\bar{Q}}_{>}^{c}$. Notice that *T*_<_always decreases very slowly with *β*_1 _(see also Figure 3(a)), showing that as *β*_1 _increases, less amount of initial cancer cells is needed to trigger a cancer. But this variation is quite insensitive.

**Figure 5:**
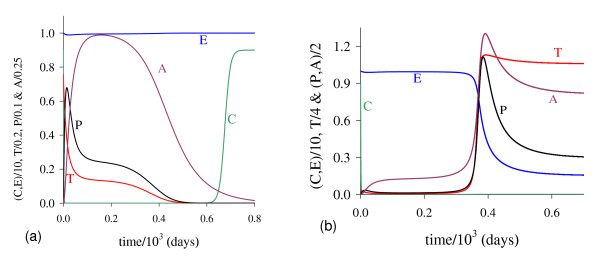
**Dynamical trajectories using *β***_**1 **_**= 10.0**. Dynamical trajectories of the system (1) considering the values of parameters given in Table 2, except *β*_1 _= 10.0. The initial conditions determine the region of attraction: trivial ${\bar{Q}}^{0}$ for *T*(0) = 0.151 (a), or non-trivial ${\bar{Q}}_{>}^{*}$ for *T*(0) = 0.152 (b). The scales of vertical and horizontal axes must be multiplied by the factors shown in the legends to obtain the actual values.

In Figure 5 we show the dynamical trajectories, changing only *β*_1 _in 1000 times the values of parameters given in Table 2, or *β*_1 _= 10.0, being attracted to the special equilibrium point ${\bar{Q}}^{c}$, which is biologically feasible, but does not describe evolution of cancer. Dynamical trajectories are attracted to ${\bar{Q}}^{0}$ (a) when *T*(0) = 0.151, while for *T*(0) = 0.152, to ${\bar{Q}}_{>}^{c}$ (b). The dynamical trajectories in Figure 5 are similar to Figure 1, except *C*, which decreases abruptly to zero and situates practically in the vertical axis, and after 650 *days *increases quickly to $\bar{C}$ (a), or remains in the horizontal axis (b). Increasing *β*_1 _in 100-fold, *T*(0) decreased 7-fold. For another *β*_1 _= 100.0, when *T*(0) = 0.142, trajectories go to ${\bar{Q}}^{0}$; while for *T*(0) = 0.143, to ${\bar{Q}}_{>}^{c}$, which coordinates have exactly the same values found in the previous case (*β*_1 _= 10.0). Both cases (figures not shown) correspond to $\beta_{1}>\beta_{1}^{c}$, hence ${\bar{Q}}_{>}^{c}$ are the same, while ${\bar{Q}}_{<}^{c}$ decrease little bit.

Let us assess the normal cells affecting negatively in the cancer cells, by varying *β*_2_.

**Figure 6:**
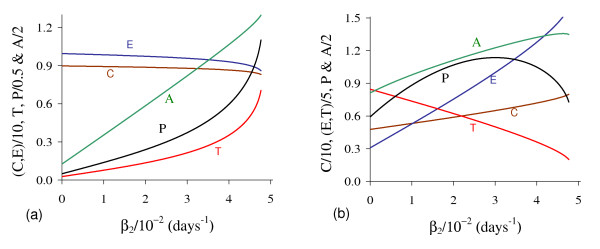
**Positive equilibrium varying *β***_**2**_. The coordinates of the positive equilibrium points varying *β*_2_. In (a) we show the coordinates of the small equilibrium point ${\bar{Q}}_{<}^{*}$, and in (b), of the big equilibrium point ${\bar{Q}}_{>}^{*}$. The small root *T*_<_increases, while the big one *T*_> _decreases up to $\beta_{2}^{c}$, at which *T*_<_= *T*_<_, and then *f*(*T*) = *g*(*T*) does not have positive solution. The scales of vertical and horizontal axes must be multiplied by the factors shown in the legends to obtain the actual values.

In Figure 6 we show the coordinates of the equilibrium points ${\bar{Q}}_{<}^{*}$ (a) and ${\bar{Q}}_{>}^{*}$ (b) by varying *β*_2_. Positive solutions for equation (5) disappeared for higher *β*_2_.

**Figure 7:**
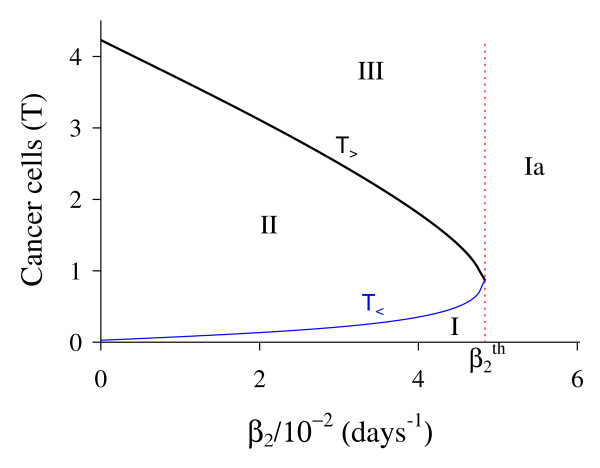
**Bifurcation diagram varying *β***_**2**_. The bifurcation diagram of $\bar{T}$ varying *β*_2_, showing the attracting regions. With respect to the coordinates of small equilibrium point ${\bar{Q}}_{<}^{*}$ for $\beta_{2}<\beta_{2}^{th}$, the trivial ${\bar{Q}}^{0}$ is attractor for initial conditions in region I, and for regions II and III, trajectories are attracted to ${\bar{Q}}_{>}^{*}$. For $\beta_{2}>\beta_{2}^{th}$, all initial conditions (region Ia) are attracted to ${\bar{Q}}^{0}$. The scales of vertical and horizontal axes must be multiplied by the factors shown in the legends to obtain the actual values.

In Figure 7, we show the bifurcation diagram, considering $\bar{T}$ as a function of *β*_2 _(curve *T *in (a) and (b) of Figure 6). For $\beta_{2}<\beta_{2}^{th}$, initial conditions set in region marked with I are attracted to the trivial equilibrium point ${\bar{Q}}^{0}$, while for those set in regions II and III are attracted to big non-trivial equilibrium point ${\bar{Q}}_{>}^{*}$. However, for $\beta_{2}>\beta_{2}^{th}$ all initial conditions are attracted to ${\bar{Q}}^{0}$, which is the unique equilibrium, because there is not any positive solution for equation (5). Hence, there is a threshold of the parameter *β*_2_, denoted by $\beta_{2}^{th}$, above which all trajectories go to trivial equilibrium. At the threshold value $\beta_{2}^{th}=4.8376\times 10^{-2}$ both roots assume same value, that is, $T_{<}\left(\beta_{2}^{th}\right)=T_{>}\left(\beta_{2}^{th}\right)=0.7053$.

**Figure 8:**
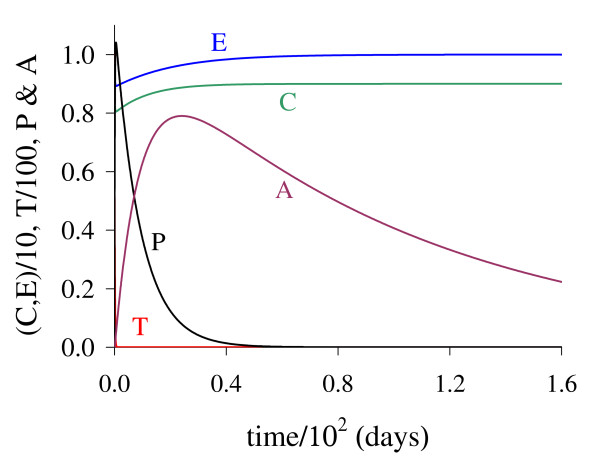
**Dynamical trajectories using *β***_**2 **_= **0.04**. Dynamical trajectories of the system (1) considering the values of parameters given in Table 2, except *β*_2 _= 0.04 (near $\beta_{2}^{th}$). The initial conditions determine the region of attraction: trivial ${\bar{Q}}^{0}$ for *T*(0) = 10.60 (a), or non-trivial ${\bar{Q}}_{>}^{*}$ for *T*(0) = 10.61 (b). The scales of vertical and horizontal axes must be multiplied by the factors shown in the legends to obtain the actual values.

For $\beta_{2}<\beta_{2}^{th}$ the dynamical trajectories are similar to that shown in Figure 1. For instance, when *β*_2 _= 0.04, for *T*(0) = 10.60 trajectories are attracted to ${\bar{Q}}^{0}$; while for *T*(0) = 10.61, to ${\bar{Q}}_{>}^{*}$. Notice that increasing *β*_2 _in 4-fold, *T*(0) increased 10-fold, showing that negative influence on cancer cells by normal cells affects strongly (very sensitive) in the cancer dynamics. For $\beta_{2}>\beta_{2}^{th}$, all initial conditions are attracted to trivial disregarding initial conditions. Figure 8 shows an extreme example, changing only *β*_2 _in 100 times the values of parameters given in Table 2, or *β*_2 _= 1.0. In this simulation we consider a very high (unrealistic) initial conditions *T*(0) = 100.0, even though ${\bar{Q}}^{0}$ is the attractor: we observe that *T *decreases quickly (practically in the vertical axis), and *A *decreases slowly. We stress the fact that we used as the initial condition for *C*, the equilibrium value of normal cells, that is, *C*(0) = *C*_0 _= 9.0, because the amount *C*_*m *_in the initial condition for *T *is higher than *C*_0_.

As *β*_1 _increases, the initial number of cancer cells (for instance, originated by mutation) needed to trigger a cancer *T*(0) = *C*_*m *_is decreased, but smoothly. Also, the normal cells can be displaced by cancer cells for values of *β*_1 _higher than its critical value, that is, $\beta_{1}>\beta_{1}^{c}$. With respect to *β*_2_, we observed a threshold for $\beta_{2},\beta_{2}^{th}$, above which cancer can not be settled. Also, as *β*_2 _increases, the initial number of cancer cells needed to trigger a cancer *C*_*m *_increases quickly, avoiding the process of cancer disease. Therefore, cancer can be settled in an organ if the following combination matches: better fitness of cancer cells (*β*_1 _increases), and decrease in the efficiency of the repairing systems (*β*_2 _decreases).

#### Recruitment of existing epithelial cells - *γ*

New network of blood vessels is created by cancer cells to provide nutrients and oxygen to support their growth. This new network depends on the capacity of originating sprouts from the existing blood vessel network, and is described by the parameter *γ*, which is varied.

**Figure 9:**
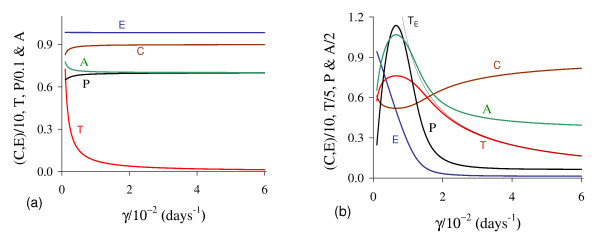
**Positive equilibrium values varying *γ***. The coordinates of the positive equilibrium points varying *γ*. In (a) we show the coordinates of the small equilibrium point ${\bar{Q}}_{<}^{*}$, and in (b), of the big equilibrium point ${\bar{Q}}_{>}^{*}$. In (b) we also show the curve of *T*_*E*_, which is always greater than *T*_>_. The small root *T*_<_decreases, while the big one *T*_> _decreases after an initial increase. The scales of vertical and horizontal axes must be multiplied by the factors shown in the legends to obtain the actual values.

In Figure 9 we show the coordinates of the equilibrium points ${\bar{Q}}_{<}^{*}$ (a) and ${\bar{Q}}_{>}^{*}$ (b) by varying *γ*. The small *T*_<_decreases, while the big *T*_>_decreases after an initial increase. Notice that *T*_>_decreases due to the fact that pre-existing blood vessels *E *decreases quickly. The new blood vessels *A *also decrease. The maximum of the variables occurs at around *γ *= 6.57 × 10^-3 ^(for *C *is minimum). In Figure 9(b) we show the curve *T*_*E*_, which depends on *γ *and situates always above the equilibrium value *T*_>_. Hence, we have non-trivial equilibrium point ${\bar{Q}}^{*}$ for sufficiently higher values of *γ*.

**Figure 10:**
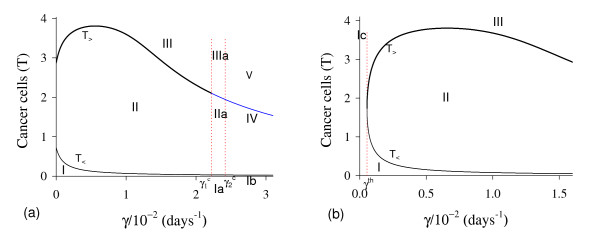
**Bifurcation diagram varying *γ***. The bifurcation diagram of $\bar{T}$ varying *γ*, showing the attracting regions. In (a) we show wide range of variation of *γ*, and in (b), a zoom near origin. For *γ *<*γ*^*th *^(b) the trivial ${\bar{Q}}^{0}$ is attractor for all initial conditions (region Ic). For *γ *> *γ*^*th *^(a), with respect to the coordinates of small equilibrium point ${\bar{Q}}_{<}^{*}$, the trivial ${\bar{Q}}^{0}$ is attractor for regions I, Ia and Ib; and we have three possibilities: (1) for $\gamma^{th}<\gamma <\gamma_{1}^{c}$, the non-trivial ${\bar{Q}}_{>}^{*}$ is attractor for initial conditions in regions II and III; (2) for $\gamma_{1}^{c}<\gamma <\gamma_{2}^{c}$, stable limit cycle circulating unstable ${\bar{Q}}_{>}^{*}$, in regions IIa and IIIa; and (3) for $\gamma >\gamma_{2}^{c}$, trivial ${\bar{Q}}^{0}$ is attractor for regions IV and V. In the latter case, the way to reaching the trivial equilibrium is different for initial conditions in Ib and IV or V. The scales of vertical and horizontal axes must be multiplied by the factors shown in the legends to obtain the actual values.

In Figure 10, we show the bifurcation diagram, considering $\bar{T}$ as a function of *γ *(curve *T *in (a) and (b) of Figure 9, which appear at *γ *= *γ*^*th*^). In (b), a zoom near zero is shown. In Figures 10(a) and 10(b), for *γ *>*γ*^*th *^initial conditions set in regions marked with I, Ia and Ib are attracted to the trivial equilibrium point ${\bar{Q}}^{0}$. However, initial conditions set in regions marked with II and III are attracted to ${\bar{Q}}_{>}^{*}\left(\gamma^{th}<\gamma <\gamma_{1}^{c}\right)$; to a limit cycle circulating ${\bar{Q}}_{>}^{*}$ in regions IIa and IIIa $\left(\gamma_{1}^{c}<\gamma <\gamma_{2}^{c}\right)$; and to ${\bar{Q}}^{0}$ in regions IV and V $\left(\gamma >\gamma_{2}^{c}\right)$, where *E *decreases and, then, increases to equilibrium value *E*_0_. The Hopf bifurcation occurs at $\gamma =\gamma_{1}^{c}$ (supercritical) and $\gamma =\gamma_{2}^{c}$ (subcritical) [17]. The special values are: *γ*^*th *^= 5.450 × 10^-4 ^and $T_{>}\left(\gamma^{th}\right)=1.74,\gamma_{1}^{c}=2.3196\times 10^{-2}$ and $T_{>}\left(\gamma_{1}^{c}\right)=2.1029$, and $\gamma_{2}^{c}=2.5161\times 10^{-2}$ and $T_{>}\left(\gamma_{2}^{c}\right)=1.9443$. Figure 9(b) showed that at $\gamma ≃\gamma_{1}^{c}$, *E *is very low. For *γ < γ*^*th *^(b), all initial conditions set in region marked with Ic are attracted to the trivial equilibrium point ${\bar{Q}}^{0}$, which is the unique equilibrium, because there is not any positive solution for equation (5). Hence, there is a threshold of the parameter *γ*, denoted *γ*^*th*^, below which all trajectories go to trivial equilibrium.

In cancer disease, it is expected that a small number of epithelial cells must be recruited in order to build up new vascularization from the sproutings. For small values of *γ *but higher than the threshold (*γ > γ*^*th*^), the dynamical trajectories follow those shown in Figure 1. In Appendix C we illustrate the Hopf bifurcation, which behavior is not compatible with real cancer. Considering number of tumor cells, concentration of growth factor and volume of blood vessels feeding the tumor, Agur *et al*. [18] showed that Hopf bifurcation can not occur if ordinary differential equations are used. But, Hopf bifurcation can occur if time-delay is encompassed. Our model presented Hopf bifurcation, however only in a range of values of parameter *γ *which is not compatible with biological findings.

Solid tumors need extra source of resources to attend the quick growth of cancer cells. Hence, cancer can be settled in an organ if the capacity of sprouting from existing vascularization is sufficiently higher (*γ *>*γ*^*th*^). However, it must not be so higher in order to avoid the death of the cancer diseased person due to normal cells being displaced by cancer cells quickly.

#### Capacity of building up new vascularization - *ε*

The appearance of shunts from existing blood vessels to initiate new vascularizations was described by the parameter *γ*. New network of blood vessels is created by cancer cells to provide nutrients and oxygen to support their growth. After a period of time *δ*^-1^, new vessels are built up from the shunts. The capacity of mounting up new vessels by cancer cells is analyzed by varying *ε*.

**Figure 11:**
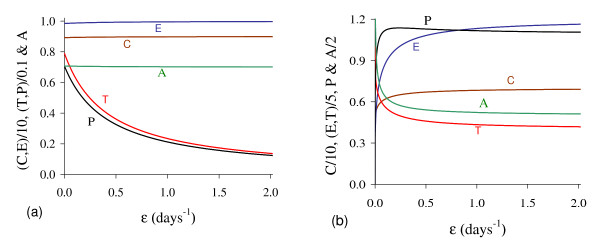
**Positive equilibrium values varying *ε***. The coordinates of the positive equilibrium points varying *ε*. In (a) we show the coordinates of the small equilibrium point ${\bar{Q}}_{<}^{*}$, and in (b), of the big equilibrium point ${\bar{Q}}_{>}^{*}$. The coordinates of ${\bar{Q}}_{<}^{*}$ and ${\bar{Q}}_{>}^{*}$ decrease. The scales of vertical and horizontal axes must be multiplied by the factors shown in the legends to obtain the actual values.

In Figure 11 we show the coordinates of the equilibrium points ${\bar{Q}}_{<}^{*}$ (a) and ${\bar{Q}}_{>}^{*}$ (b) by varying *ε*. Both small *T*_*<*_and big *T*_*>*_decrease. In (b), due to the solution *T*_*>*_(*ε *= 0) = 5.0, we used equation (12) for *f*(*T*) and *g*(*T*) to obtain *Ā*(*0*) = 4.457.

**Figure 12:**
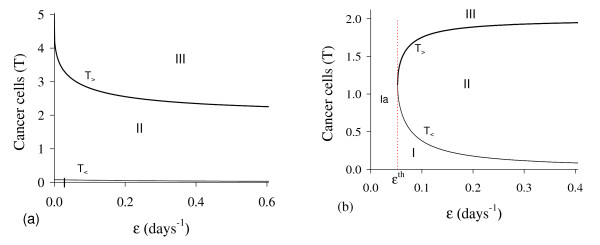
**Bifurcation diagram varying *ε***. The bifurcation diagram of $\bar{T}$ varying *ε*, showing the attracting regions. With respect to the coordinates of small equilibrium point ${\bar{Q}}_{<}^{*}$, the trivial ${\bar{Q}}^{0}$ is attractor for initial conditions in a very small region I, and for initial conditions in regions II and III ${\bar{Q}}_{>}^{*}$ is the attractor (a). There is not a critical value due to influx *γP *in equation for *A*. However, for *γ *= 0, we have bifurcation diagram similar to the Figure 10.b: for *ε *<*ε*^*th*^, trivial ${\bar{Q}}^{0}$ is attractor in I (and for all initial conditions in Ia), and ${\bar{Q}}_{>}^{*}$ is attractor in II and III (b) for *ε *> *ε*^*th*^. The scales of vertical and horizontal axes must be multiplied by the factors shown in the legends to obtain the actual values.

In Figure 12, we show the bifurcation diagram, considering $\bar{T}$ as a function of *ε *(curve *T *in (a) and (b) of Figure 11). When *γ >*0, there are not neither special nor threshold values: when initial conditions are set in a small region I, trajectories are attracted to the trivial equilibrium point ${\bar{Q}}^{0}$, and for initial conditions set in regions II and III, trajectories are attracted to ${\bar{Q}}_{>}^{*}$ (a). This behavior results by the existence of influx in equation for *A*, given by the term *γP*. Hence, if we let *γ *= 0, a different bifurcation arises (b).

**Figure 13:**
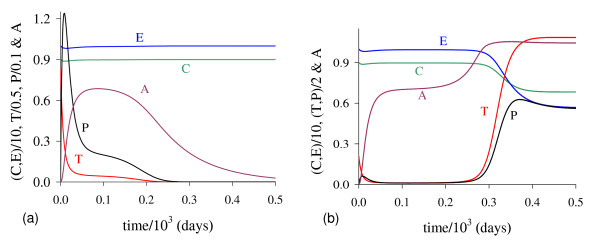
**Dynamical trajectories using *ε *= 1.0**. Dynamical trajectories of the system (1) considering the values of parameters given in Table 2, except *ε *= 1.0. The initial conditions determine the region of attraction: trivial ${\bar{Q}}^{0}$ for *T*(0) = 0.416 (a), or non-trivial ${\bar{Q}}_{>}^{*}$ for *T*(0) = 0.417 (b). The scales of vertical and horizontal axes must be multiplied by the factors shown in the legends to obtain the actual values.

There is a threshold of *ε*, denoted *ε*^*th*^, below which all trajectories go to trivial equilibrium. For *ε > ε*^*th*^, where *ε*^*th *^= 5.250 × 10-^2^, we have similar behavior than that observed in (a), but *T*_>_is increasing. In Figure 13 we show the dynamical trajectories depending on the initial conditions for *γ *= 0, changing only *ε *in 100 times the values of parameters given in Table 2, or *ε *= 1.0. Dynamical trajectories are attracted to ${\bar{Q}}^{0}$ (a) when *T*(0) = 0.416, while for *T*(0) = 0.417, to ${\bar{Q}}_{>}^{*}$ (b). The dynamical trajectories in (a) are similar to Figure 1(a). Increasing *ε *in 100-fold, *T*(0) decreased 2.5-fold.

Due to the influx *γP*, which is a linear term, in the equation for *A*, the parameter *ε *that describes the mounting up of new vascularization does not present any special behavior, except by the dependency with the initial conditions. However, when *γ *= 0, there arises a threshold of *ε*, called *ε*^*th*^, above which new vascularizations promoted by cancer cells can occur. The case *γ *= 0 means that cancer cells are supported exclusively by the new network of blood vessels originated from surrounding tissues for instance, and the pre-existing one maintains its function of nourishing exclusively the normal cells.

Angiogenesis is the process by which new blood vessels develop from an existing vasculature, through endothelial cell sprouting, proliferation, and fusion. Hence, angiogenesis create new vascularization from sprouting originated in existing vasculature, which was called pre-angiogenesis. Due to the endothelial cell sprouting promoted by the vascular endothelial growth factor, cancer cells can grow even in the absence of new vascularization (*ε *= 0), being the size of cancer cells big with higher capacity of mounting up new vascularization (increasing *ε*).

## Discussion

In foregoing section we have used for *k*_3 _and *k*_4 _values comparable to *k*_1 _and *k*_2 _in order to enhance the results. In other words, cancer related cells are allowed to grow comparable to the size of normal cells, which is not true. In real world, cancer related cells are found in much smaller size, hence *k*_3 _and *k*_4 _must be lower than *k*_1 _and *k*_2_, being the relative size depending on the organ of the body.

**Figure 14:**
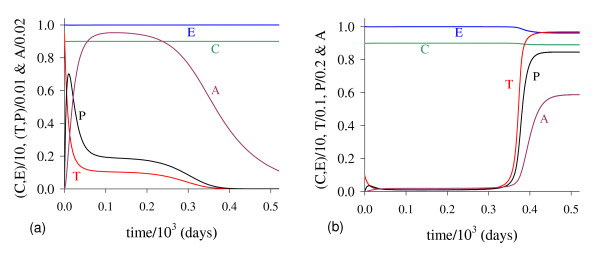
**Dynamical trajectories using alternative values**. Dynamical trajectories of the system (1) considering the fixed values of parameters given in Table 2, except those in the column marked with **. The initial conditions determine the region of attraction: trivial ${\bar{Q}}^{0}$ for *T*(0) = 9.51 × 10^-3 ^(a), or non-trivial ${\bar{Q}}_{>}^{*}$ for *T*(0) = 9.52 × 10^-3 ^(b). The scales of vertical and horizontal axes must be multiplied by the factors shown in the legends to obtain the actual values.

In Figure 14 we show dynamical trajectories using the initial conditions given in equation (2). The values of the parameters are given in the column marked with ** of Table 2, and other parameters are those given in fixed values. Dynamical trajectories are attracted to ${\bar{Q}}^{0}$ (a) when *T*(0) = 9.51 × 10^-3^, while for *T*(0) = 9.52 × 10^-3^, to ${\bar{Q}}_{>}^{*}$ (b). The cancer is triggered at around 400 *days*. Decreasing *k*_3 _in 50-fold (and some values of other parameters were also changed), the initial value that divides two attracting regions *T*(0) is around 100-fold lower than that shown in Figure 1. In Figure 1, *T *reaches the asymptotic value near *k*_3 _= 5.0, while in Figure 14, near *k*_3 _= 0.1. Then all previous simulations can be translated to real world by appropriate scaling factors.

The initial amount of cancer cells originated from normal cells by mutations plays an important role in the dynamics of cancer growth. When the interacting parameters *β*_1_, *γ *and *ε *increase, the small solution *T*_<_decreases. This decrease in the initial cancer cells necessary to trigger the cancer is mediated by cancer cells that: (1) inhibit and occupy normal cells habitat (*β*_1_); (2) produce higher amount of substances which cause new blood vessels to grow (*γ*); and (3) construct new blood networks to nourish themselves (*ε*). In opposite way, when the interacting parameter *β*_2 _increases, the small solution *T*_<_also increases. Hence, when normal cells inhibit the growth of cancer cells by many factors as better fitness, increasing repairing action, and induction of apoptosis, then the onset of cancer is avoided.

Another important aspect in the cancer growth is the appearance of thresholds. The interacting parameters *β*_2 _and *γ *present threshold values, respectively, $\beta_{2}^{th}$ and *γ*^*th*^. Usually the systems that control the production of substances that induce the formation of new blood vessels to grow operate normally, which have as consequence that cancer cells are unable to recruit the blood to supply their need to continue to proliferate, and they fade out at this early stage. However, cancer cells may begin to produce substances which cause new blood vessels to grow. This phenomenon is characterized by the threshold of *γ*. The threshold of *β*_2 _can be understood as the well functioning repairing mechanisms and the low fitness of cancer cells in comparison with normal cells. The threshold of *ε *arises only for *γ *= 0, which mimics cancer cells being nourished only by the new network of blood vessels originated from surrounding tissues by vasculogenesis [14].

There are also critical values for parameters *β*_1 _and *γ*. Critical value for *β*_1 _is a mathematical artefact, because it is meaningless biologically (in general *k*_3 _is very low in comparison with constraint *T*_*E*_). With respect to *γ*, there are two critical values, named $\gamma_{1}^{c}$ and $\gamma_{2}^{c}$. When *γ *increases, the epithelial cells *E *decrease, and damped oscillations appear. When *γ *approaches to $\gamma_{1}^{c}$, the oscillations are less damped, and when surpasses $\gamma_{1}^{c}$, regular oscillations occur. However, the amplitude of regular oscillations increases as *γ *approaches to $\gamma_{2}^{c}$, resulting for lowest values of *E, T *and *A *reaching zero values (see figures in Appendix C). When the lowest values are incapable to trigger new burst of cancer cells, the oscillations cease and the trivial is the attractor. This occurs when *γ *surpasses $\gamma_{2}^{c}$. Again both $\gamma_{1}^{c}$ and $\gamma_{2}^{c}$ do not bear any biological meanings, because the cure of cancer is due to the elimination of pre-existing network of blood vessels. The biological meaningless sustained oscillations begin at $\gamma =\gamma_{1}^{c}$ (the supercritical Hopf bifurcation) and cease at $\gamma =\gamma_{2}^{c}$ (the subcritical Hopf bifurcation).

For instance, considering values of Table 2, the column with **, we observe the same behavior than that found in figures shown in Appendix C as *γ *increases: (1) damped oscillations around ${\bar{Q}}^{*}$ for *γ *= 0.75; (2) regular oscillations around ${\bar{Q}}^{*}$ in the interval [0.76,0.80]; and (3) to the trivial equilibrium ${\bar{Q}}^{0}$ for *γ *= 0.81. In the region of limit cycle, we observed that: (1) *C *oscillates between (8.9, 9.0) for all *γ*; *E, P *and *A *oscillate between (0*, M*), where *M *increases as *γ *increases; and (3) *T *oscillates between (*m,*0.1), where *m *decreases as *γ *increases. When *m *becomes very small, there is not burst of cancer cells, limit cycle is destroyed, and cancer fades out for higher values of *γ*. Notice that the amplitude of oscillations of normal cells *C *is not affected, in opposite way of epithelial cells *E *which drops to near 0.

Agur *et al*. [18] showed that ordinary differential equations admit Hopf bifurcation if and only if at least one time-delay is introduced in the tumor growth modeling. They then concluded that an appropriate candidate for describing the cancer growth is the alternative that includes time-delays in the tumor proliferation or angiogenesis process. They also concluded that further mathematical research is warranted for exploring time-delays in the biologically realistic domains in the parameter spaces. Our model, however, showed Hopf bifurcation without time-delay (in fact, there is an elapse of time between pre-angiogenesis and angiogenesis cells), and sustained oscillations occur only in biologically not realistic domains in the parameter space.

When the initial conditions, especially *T*(0) = *C*_*m*_, are such that the non-trivial equilibrium is attractor, the cancer cells reach the level *T*_>_. *C*_*m *_increases with increasing *β*_1_, decreases with *β*_2 _and *ε*. Cancer cells can grow and reach higher levels when they affect negatively normal cells (*β*_1_), but reach lower levels when normal cells acts as a barrier against them (*β*_2_). When *γ *varies, *T*_>_increases in the initial phase (*E *decreases), and then decreases (*E *is practically zero). The parameter *γ *plays an equivalent role of *β*_1_, but, restricted only to *E*, which decreases it dramatically. For lower *γ*, there is sufficient number of *E *to increase *A*, but epithelial cells are exhausted as *γ *increases, and *A *decreases (see equation (4)). Finally, *T*_>_decreases with *ε*, which comes out due to relative higher value of *γ*. The behavior of *ε *is strongly dependent on *γ *due to the influx *γP *in equation for *A: *for lower *γ *(also *γ *= 0), *T*_>_increases with *ε *(see Figure 12(b)).

**Figure 15:**
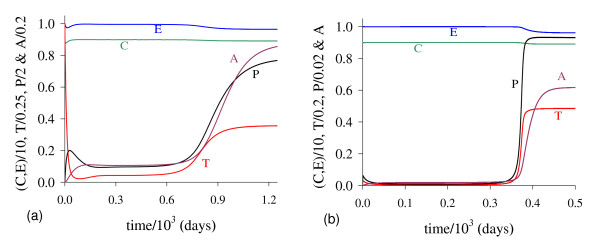
**Dynamical trajectories using *δ *= 0.001 and *δ *= 10.0**. Dynamical trajectories of the system (1) considering the fixed values of parameters given in Table 2, except those in the column marked with **. The initial conditions determine the region of attraction. For *δ *= 0.001 non-trivial ${\bar{Q}}^{*}$ is attractor when *T*(0) = 2.493 *× *10^-1 ^(a), while for *δ *= 10.0, when *T*(0) = 6.609 *× *10^-3 ^(b). The scales of vertical and horizontal axes must be multiplied by the factors shown in the legends to obtain the actual values.

We introduced in the model an intermediate phase between epithelial cells and angiogenesis cells. The purpose was to consider a delay in new blood vessel formation (angiogenesis) by the period of time *δ*^-1^. This can be suppressed by letting *δ *→ ∞. In Figure 15 we show dynamical trajectories using the initial conditions given in equation (2), and values of the parameters given in the column marked with ** of Table 2, and other parameters are those given in fixed values, except *δ*. Dynamical trajectories are attracted: (1) for *δ *= 0.001, to ${\bar{Q}}^{0}$ (not shown) when *T*(0) = 2.492 × 10^-1^, while for *T*(0) = 2.493 × 10^-1^, to ${\bar{Q}}_{>}^{*}$ (a); and (2) for *δ *= 10.0, to ${\bar{Q}}^{0}$ (not shown) when *T*(0) = 6.608 × 10^-3^, while for *T*(0) = 6.609 × 10^-3^, to ${\bar{Q}}_{>}^{*}$ (b). The cancer is triggered at around 900 and 360 *days*, respectively for *δ *= 0.001 and 10.0. Including Figure 14(b), the cancer trigger is delayed and initial cancer formation due to mutation must be increased as *δ *decreases.

## Conclusions

Many models [5][19] have already been proposed to describe cancer growth, and some of those models have explicitly considered the spatial dimension [20-22], which has been shown to play a key role in the understanding of various tumor growth processes. Other models considered computational approach [23,24]. Spatial and computational modelings were analyzed numerically. However, we developed a zero-dimensional model for the initial stages of tumor angiogenetic growth in order to obtain analytical results.

In this model, various cell species are supposed to satisfy Lotka-Volterra growth laws. Neither metastasis nor geometry of the solid cancer were taken into account. The initial conditions (2), supplied to the dynamical systems (1), describe an impulsive system: in a steady state, a perturbation is introduced at a time *t *= *t*_0 _= 0 as a form of pulse (Dirac delta function). This pulse mimics normal cells mutating to cancer cells. Cancer cells then promote the mounting of new network of blood vessels to nourish them after an elapse of time *δ*^-1^. We introduced the pre-angiogenesis cells *P *to include the time delay in order to completing functional angiogenesis.

From the model, we conclude that the dynamical trajectories depend on the initial conditions supplied to the system, and also on interacting parameters. The cumulative effects of mutation is essential to originate a cancer cell. This effect is captured by the initial amount of cancer cells originating from normal cells, denoted by *C*_*m*_. A sufficient number of cells must suffer mutation in order to a concentration of *C*_*m *_cells really bear all necessary mutations to become effectively cancer cells. Our model is spatially homogeneous, hence the initial number of cancer cells is *C*_*m *_× *V*, where *V *is the volume of an organ of human body. In extremely favorable environmental and individual conditions, this initial number can be one.

Initially, cancer cells always can grow. But they fade out if they are unable to build up new blood vessels in order to supply their needs. The capacity of inducting new vascularization from existing blood vessel network must be efficient (*γ > γ*^*th*^), and better fitness (increasing capacity of proliferation and capturing nutrients, decreasing mortality, etc.) of cancer cells (*β*_1 _increases) in comparison with normal cells. Another aspect of cancer growth is corruption of the repairing systems, and in some extend we can think of that normal cells influencing negatively cancer cells play the role of repairing (fixing mutated DNA and inducting apoptosis). The parameter *β*_2 _measures the efficient action of repairing system, and the effect of decreasing this value result in a higher level of corruption in the repairing system. Since cancer cells can recruit epithelial cells to form new blood vessels, the capacity of proliferation of new vessels (*ε*) does not present threshold value.

The parameter *γ *can be thought of deviation of nutrients and oxygen from normal cells to feed cancer cells. This deviation does not affect normal cells because the carrying capacity of normal cells *k*_1 _does not depend on the size of network of blood vessels. As *γ *increases, the deviated network plus the new one mounted by the action of angiogenesis effectors nourish the cancer cells. Hence, *γ *= 0 means that pre-existing network of blood vessels feeds normal cells, while the new network nourishes cancer cells. In this case, the capacity of cancer cells in promoting new vascularization is essential, which must surpass the threshold value *ε*^*th*^. We obtained biologically feasible non-trivial equilibrium points, that is, the coordinates of the equilibrium points are positively defined. However, due to the simplifications assumed by model, the range of variations of parameters like *β*_1 _and *γ *must be restricted. When $\beta_{1}>\beta_{1}^{c}$ we have an equilibrium point ${\bar{Q}}^{c}$ with $\bar{C}=0$, while for $\gamma_{1}^{c}<\gamma <\gamma_{2}^{c}$, we have limit cycle (sustained oscillations) with *E *~ 0, but for $\gamma >\gamma_{2}^{c}$, we have abrupt increase of *E*, and the trivial equilibrium is attained. Both results can not be acceptable for cancer growth description.

Some results obtained here can be understood as vasculogenesis (when *γ *= 0) [14]. The dynamics of normal and cancer cells are similar than that presented in the model proposed by Nani and Freedman [11]. However, we did not take into account the action of immune system, while they did not take into account the angiogenesis. Agur *et al*. [18] proposed to examine the occurrence of Hopf bifurcation in the clinical context, that is, to check whether or not one can contain tumor growth by imposing time-delays in the processes of neo-vascularization. Our results showed that Hopf bifurcation occurs in biologically not realistic domains in the parameter space.

In a future work we will analyze a model in which the sizes of the normal and cancer cells are allowed to depend on the overall network of blood vessels: normal and cancer cells compete for nutrients provided by the pre-existing blood vessels, while cancer cells have additional source originated from angiogenesis. If we take these effects into account in the model, maybe Hopf bifurcation can be avoided. There are several ways to improving model given in equation (1). One is the dependency of normal cells with the size, which decreases with increasing *γ*, of the existing vasculature.

For instance, we can deal with intermittent process instead of a continuous process of sprouting from existing blood vessels. We can change the second and fourth equations by

$$\left\{\begin{array}{c} \frac{d}{dt}E=\alpha_{2}E\left(1-\frac{E}{k_{2}}\right)-\Phi \left(t\right)=\mu_{2}E\\ \frac{d}{dt}P=\Phi \left(t\right)-\delta P-\mu_{4}P, \end{array}\right)$$

where Φ(*t*) is the total intermittent sproutings rate. One form of Φ(*t*) is

$$\Phi \left(t\right)=\overset{n}{\underset{i=0}{\sum }}\gamma_{i}\theta \left(t-\tau_{i}\right)\theta \left(\tau_{i+1}-t\right)ET,$$

where *γ*_*i *_and *τ*_*i *_(*τ*_0 _= 0) are, for *i *= 0, ···, *n*, respectively, the *i-th *sprouting rate and the time interval during which sproutings occur. The Heaviside function *θ*(*x*) is such that *θ*(*x*) = 1 if *x *> 0, and *θ*(*x*) = 0, otherwise. To be intermittent, we must have *γ*_2*i *_> 0 and *γ*_2*i*+1 _= 0, that is, sproutings occur in the time interval [0, *τ*_1_] with rate *γ*_0_, do not occur in the interval [*τ*_1_*, τ*_2_], and so on. Another possibility is

$$\Phi \left(t\right)=\overset{n}{\underset{i=0}{\sum }}s_{i}\delta \left(t-\tau_{i}\right)E,$$

where *s*_*i *_and *τ*_*i *_are, for *i *= 0, ···, *n*, respectively, the *i-th *proportion of epithelial cells generating sproutings and the time at which sproutings occur. The proportion *s*_*i *_can depend on *T*. Vaccination campaigns against viral infections were analyzed considering age interval vaccination (Heaviside function) [25] or a series of pulses (Dirac function) [26].

Another improvement of the model (1) is the introduction of the immune response. This can be done by introducing lymphocyte cells action as Nani and Freedman dealt with [11].

In the model (1), chemotherapy that acts specifically against tumor cells can be introduced easily. An intermittent chemotherapy can be introduced in the model by adding one term in the first equation, that is,

$$\frac{d}{dt}T=\alpha_{3}AT\left(1-\frac{T}{k_{3}}\right)-\beta_{2}CT-\mu_{3}T-\mu_{Q}\frac{TQ}{k_{q}+Q},$$

and adding an equation for the drug administration as

$$\frac{d}{dt}Q=q\left(t\right)-l\mu_{Q}\frac{TQ}{k_{q}+Q}-\mu_{q}Q,$$

where *Q *is the concentration of drug at time t, and *μ*_*Q *_and *μ*_*q *_are the rates of, respectively, intake of drug by cancer cells and elimination of drug by body. The parameter *l *is the amount of drugs intake by one cancer cells, and *q*(*t*) is the drug administration rate. The kinetics of drug intake follows Michaelis-Menten [27]. If *q*(*t*) = *q*, a fixed value, we have a continuous regimen of administration. Intermittent drug administration can be considered. First, we can define

$$q\left(t\right)=\overset{n}{\underset{i=0}{\sum }}u_{i}\delta \left(t-\tau_{i}\right),$$

where *u*_*i *_is, for *i *= 0, ···, *n*, the *i-th *concentration of drug administered at time *τ*_*i*_. Another is

$$q\left(t\right)=\overset{n}{\underset{i=0}{\sum }}q_{i}\theta \left(t-\tau_{i}\right)\theta \left(\tau_{i+1}-t\right),$$

where *q*_*i *_and *τ*_*i *_are, for *i *= 0, ···, *n*, the *i-th *drug administration rate and the time interval during which drug is administrated. To be intermittent, we must have *q*_2*i *_*>*0 and *q*_2*i*+1 _= 0.

In this paper we obtained threshold values, but we did not deal with the effects of controlling mechanisms, which are left to a further work. Briefly, for instance, two parameters can be used in order to control cancer growth. The thresholds of the parameters *γ *and *β*_2_, named *γ*^*th *^and $\beta_{2}^{th}$, can be managed as follows.

Let us suppose that *γ *and *β*_2 _have values above and below their respective thresholds. To control cancer, parameters of the model (including dynamics of controls) must be managed to increase *γ*^*th *^to surpass *γ*, and to decrease $\beta_{2}^{th}$ to fall below *β*_2_. Another way to control cancer is acting on the probability of sproutings becoming angiogenesis cells, in order to decrease *δ*/(*δ *+ *μ*_4_), which can be done by decreasing the rate of transformation from pre-angiogenesis to angiogenesis cells *δ *and/or increasing the mortality rate *μ*_4 _of pre-angiogenesis cells.

## Competing interests

The authors declare that they have no competing interests.

## Appendix A: Non-trivial equilibrium point

**Figure 16:**
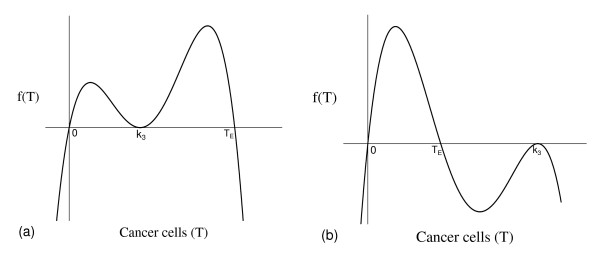
**Qualitative behavior of *f*(*T*)**. Qualitative behavior of *f*(*T*), with *k*_3 _<*T*_*E *_(a) and *k*_3 _> *T*_*E *_(b).

The non-trivial equilibrium value of cancer cells corresponding to model (1), $\bar{T}$, is the positive solution of the equation (5), that is, *f*(*T*) = *g*(*T*). The fourth degree polynomial *f*(*T*) is such that

$$\left\{\begin{array}{c} \;f\left(-\infty \right)=-\infty \\ \;f\left(0\right)=0\\ \;f\left(+\infty \right)=-\infty , \end{array}\right)$$

and has three non-negative roots: 0, (*α*_2 _- *μ*_2_)*/γ *= *T*_*E*_, and *k*_3 _= *T*_*A *_is a double root (see equation (7)). Figure 16 shows the qualitative behavior of *f*(*T*): *T*_*A *_<*T*_*E *_(a), and *T*_*A *_>*T*_*E *_(b). Notice that Figure 16(a) corresponds to higher carrying capacity for cancer cells.

The function *g*_1_(*T*) is such that

(A.1) $$\left\{\begin{array}{c} \;g_{1}\left(-\infty \right)=+\infty \\ g_{1}\left(0\right)=g_{0}=k_{3}\left[\frac{\mu_{3}}{\alpha_{3}}+\frac{\beta_{2}k_{1}}{\alpha_{3}\alpha_{1}}\left(\alpha_{1}-\mu_{1}\right)\right]>0\\ \;g_{1}\left(+\infty \right)=-\infty , \end{array}\right)$$

and has three positive roots: *ε*/*μ*_5_, *k*_3 _= *T*_*A*_, and $\frac{\alpha_{1}\mu_{3}}{\beta_{1}\beta_{2}k_{1}}+\frac{\alpha_{1}-\mu_{1}}{\beta_{1}}=T_{g}$ (see equations (7) and (8)). The function *g*_2 _(*T*) is such that

$$\left\{\begin{array}{c} g_{2}\left(-\infty \right)=-\infty \\ g_{2}\left(0\right)=0\\ g_{2}\left(+\infty \right)=+\infty , \end{array}\right)$$

and has two non-negative roots: 0, and $\frac{\alpha_{1}\mu_{3}}{\beta_{1}\beta_{2}k_{1}}+\frac{\alpha_{1}-\mu_{1}}{\beta_{1}}=T_{g}$ (double root). The dominant terms of the third degree polynomials *g*_1_(*T*) and *g*_2_(*T*), when *T *→ *± *∞, are

$$\left\{\begin{array}{ccc} g_{1}\left(T\right)=v_{1}T^{3}; & with & v_{1}=-\frac{\varepsilon \beta_{1}\beta_{2}k_{1}}{\mu_{5}\alpha_{3}\alpha_{1}}\\ g_{2}\left(T\right)=v_{2}T^{3}; & with & v_{2}\frac{\varepsilon {\left(\beta_{1}\beta_{2}k_{1}\right)}^{2}k_{3}}{\mu_{5}{\left(\alpha_{3}\alpha_{1}\right)}^{2}k_{4}}. \end{array}\right)$$

The third degree polynomial *g*(*T*) is sum of *g*_1_(*T*) and *g*_2_(*T*). Notice that, for *T *≥ 0, we have *g*_2_(*T*) ≥ 0, while *g*_1_(*T*) changes signal. Hence, the effect of *g*_2_(*T*) in the sum (*g*(*T*)) is the increasing in the values of *g*_1_(*T*), except at *T *= *T*_*g*_, at which we have *g*_1_(*T*_*g*_) = *g*_2_(*T*_*g*_). The asymptotic behavior of the polynomial *g*(*T*) depends on the velocities of increasing *v*_2 _and *v*_2 _(or coefficients) of *T*^*3*^. When *β*_1 _*β*_2 _*< α*_3 _*α*_1 _*k*_4_/(*k*_1 _*k*_3_) (generically referred to as weak interaction between normal and cancer cells when values of *β*_1 _and *β*_2 _are proportional), in which case *T*_*g *_is higher, we have

$$\left\{\begin{array}{c} g\left(-\infty \right)=+\infty \\ g\left(0\right)=g_{0}\\ g\left(+\infty \right)=-\infty . \end{array}\right)$$

It can be shown that there is one solution or three positive solutions. When *β*_1 _*β*_2 _*> α*_3 _*α*_1 _*k*_4_/(*k*_1 _*k*_3_) (generically referred to as strong interaction between normal and cancer cells when *β*_1 _and *β*_2 _are proportional), in which case *T*_*g *_is lower, we have

$$\left\{\begin{array}{c} g\left(-\infty \right)=-\infty \\ g\left(0\right)=g_{0}\\ g\left(+\infty \right)=+\infty , \end{array}\right)$$

and there is one negative solution and two positive solutions. In both cases *T*_*g *_is always a positive root of *g*(*T*), and *g*_0 _is given by equation (A.1). The existence of other positive solutions depends on the relative position of the roots of *g*_1_(*T*) and the coefficients of the polynomials.

**Figure 17:**
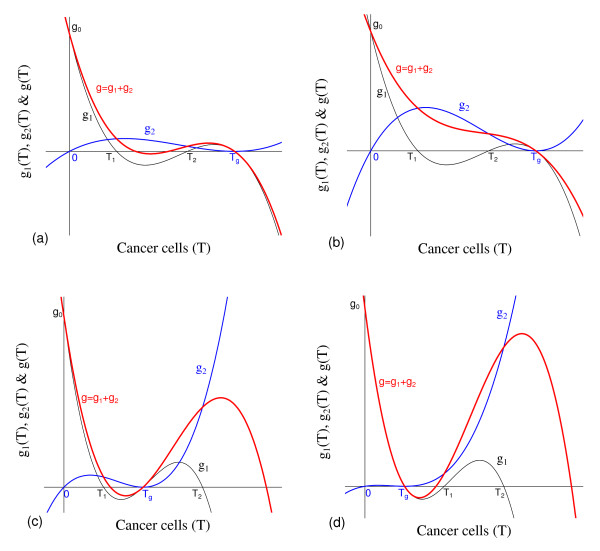
**Qualitative behavior of *g*(*T*)**. Qualitative behavior of *g*(*T*), for small *β*_1 _*β*_2 _(weak interaction): *T*_*g *_is the greatest root of *g*_1_(*T*), with three positive solutions (a), and one solution (b); *T*_*g *_is between the roots *ε*/*μ*_5 _and *k*_3_, with three positive solutions (c); and *T*_*g *_is the smallest root of *g*_1_(*T*), with three positive solutions (d). *T*_1 _and *T*_2_, the roots of *g*_1_(*T*), stand for *ε*/*μ*_5 _and *k*_3_.

Figure 17 shows the qualitative behavior of *g*(*T*) for small *β*_1 _*β*_2 _(weak interaction): when *T*_*g *_is the greatest root of *g*_1_(*T*), with three positive solutions (a), and one solution (b); when *T*_*g *_is between the roots *ε*/*μ*_5 _and *k*_3_, with three positive solutions (c); and when *T*_*g *_is the smallest root of *g*_1_(*T*), with three positive solutions (d). *T*_1 _and *T*_2_, the roots of *g*_1_(*T*), stand for *ε*/*μ*_5 _and *k*_3_, depending on the relative positions between them. Figure 17(a) and 17(b) shows clearly the effect of *g*_2_(*T*) increasing *g*_1_(*T*) and changing the roots of *g*(*T*), named $T_{1}^{g}$ and $T_{2}^{g}:T_{1}^{g}>T_{1}$ and $T_{2}^{g}<T_{2}$, and both $T_{1}^{g}$ and $T_{2}^{g}$ disappear when *g*_2_(*T*) is sufficiently higher.

The positive solution of the equation (5) is the intersection between the curves *f*(*T*) and *g*(*T*), or roots of *f*(*T*)-*g*(*T*). Notice that:

1. We do not have negative solutions, because *f*(*T*) < 0 and *g*(*T*) > 0, for *T *< 0, which implies that *f*(*T*) - *g*(*T*) < 0. At *T *= 0 we have *f*(0) - *g*(*0*) = -*g*_0 _< 0.

2. We have *f*(∞) - *g*(∞) → -∞, even when *g*(∞) → +∞, because *f*(*T*) is fourth degree polynomial, and *g*(*T*), third degree.

3. *f*(*T*) - *g*(*T*) is a continuous function, a fourth degree polynomial, where there is not solution for *T *< 0, *f*(0) - *g*(*0*) < 0 and *f*(∞) - *g*(∞) < 0. Hence, by the intermediate value theorem, we have 0, 2 or 4 positive solutions in the interval (0, ∞).

4. We are searching biologically feasible equilibrium, hence, according to equation (7), $\bar{T}$ must be lower than the lowest root *T*_*m *_of *f*(*T*), that is, $\bar{T}\in \left(0,T_{m}\right)$, where *T*_*m *_= min {(*α*_2 _- *μ*_2_)/*γ, k*_3_}, the minimum between (*α*_2 _- *μ*_2_)/*γ *and *k*_3_. Therefore, in the interval (0*, T*_*m*_) we have at most 2 positive solutions.

**Figure 18:**
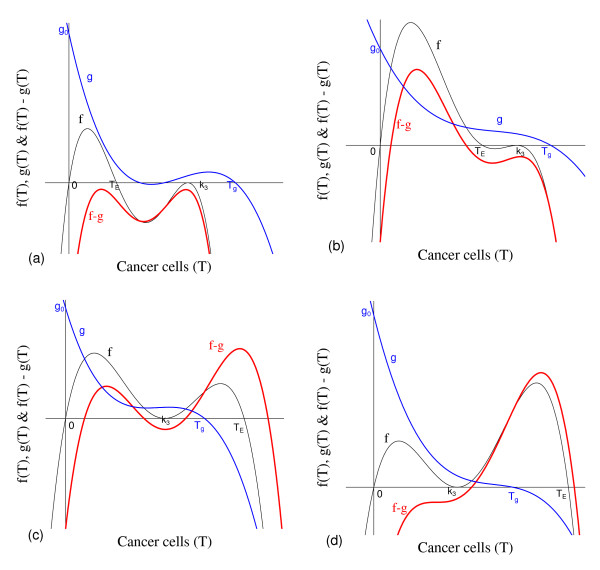
**Qualitative behavior of *f*(*T*) - *g*(*T*)**. Qualitative behavior of *f*(*T*) - *g*(*T*): none (a), two (b) and four (c) solutions. In (d), we show two positive solutions not biologically feasible, because they are greater than the constraint *T*_*A *_= *k*_3_.

Figure 18 shows qualitative behavior of *f*(*T*) - *g*(*T*). Figures 18(b) and 18(c) (first two lower roots) show two positive solutions satisfying $\bar{T}<T_{A},\bar{T}<T_{E}$ and $\bar{T}<T_{g}$. Additionally, the constraint $\bar{T}<T_{C}$ are satisfied, once $\bar{T}<T_{g}$. Hence, biologically feasible solutions are at most 2. However, Figures 18(c) (last two higher roots) and 18(d) show two positive solutions not biologically feasible, because they are greater than the constraint *T*_*A *_= *k*_3_.

When there is not any positive solution for *f*(*T*) - *g*(*T*) = 0, the unique equilibrium point is the trivial ${\bar{Q}}^{0}$. In the case of two positive solutions, the small root (*T*_*<*_) forms the unstable equilibrium ${\bar{Q}}_{<}^{*}$, and the big one (*T*_>_) forms a possibly stable (let us for simplicity say that it is stable) equilibrium ${\bar{Q}}_{>}^{*}$. Between two stable equilibria ${\bar{Q}}_{>}^{*}$ and ${\bar{Q}}^{0}$ (this always exists and is stable), we have an unstable point ${\bar{Q}}_{<}^{*}$. We call as the 'break-point' [28,29] the unstable equilibrium point ${\bar{Q}}_{<}^{*}$, which separates two attracting regions containing one of the equilibrium points ${\bar{Q}}_{>}^{*}$ and ${\bar{Q}}^{0}$. In another words, we have a hyper-surface obeying, *e.g.*, $h\left(\bar{C}\left(T_{<}\right),Ē\left(T_{<}\right),T_{<},\bar{P}\left(T_{<}\right),Ā\left(T_{<}\right)\right)=0$, generated by the coordinates of the equilibrium point ${\bar{Q}}_{<}^{*}$ such that one of the equilibrium points ${\bar{Q}}_{>}^{*}$ and ${\bar{Q}}^{0}$ is an attractor depending on the relative position of the initial conditions supplied to the dynamical system (1) with respect to the hyper-surface *h *[30].

## Appendix B: The local stability of the non-trivial equilibrium ${\bar{Q}}^{c}$

Let us show that the matrix *A*, given by equation (14), is an *M*-matrix [31] for big non-trivial equilibrium ${\bar{Q}}_{>}^{c}$, where *T*_>_is one of the coordinates.

**Definition**. We say that the *n *× *n *matrix *A *= [*a*_*ij*_] is a non-singular *M*-matrix if *a*_*ij *_≤ 0, *i *≠ *j*, and there exists a matrix *B *≥ 0 and a real number *s *> 0 such that

$$\begin{array}{ccc} A=sI-B & and & s>(\rho (B), \end{array}$$

where *I *is the identity matrix and *ρ *is the spectral radius [32].

Or, equivalently:

**Proposition 1**. *A *is a non-singular *M*-matrix if and only if the real part of its eigenvalues is greater than zero.

**Proposition 2**. *A *(elements *a*_*ij*_) is a non-singular *M*-matrix if and only if the diagonal entries are positive, and there exists a positive diagonal matrix *D *(diagonal elements *d*_*i *_> 0), such that *AD *is strictly diagonal dominant, that is,

$$a_{ii}d_{i}>\underset{j\neq i}{\sum }\left|a_{ij}\right|d_{j},$$

for *i *= 1, 2, ..., *n*.

According to the first part of Proposition 2, the matrix *A *has positive diagonal elements, see equation (14).

The second part of Proposition 2 is written as

(B.1) $$\left\{\begin{array}{c} \;\;\;\;\;\;\frac{\alpha_{2}}{k_{2}}Ēd_{1}>\gamma Ēd_{2}\\ \;\;\;\;\frac{\alpha_{3}}{k_{3}}Ā\bar{T}d_{2}>\frac{\alpha_{3}}{k_{3}}\bar{T}\left(k_{3}-\bar{T}\right)d_{1}\\ \;\;\;\left(\mu_{4}+\delta \right)d_{3}>\gamma Td_{1}+\gamma Ēd_{2}\\ \left(\delta \frac{\bar{P}}{Ā}+\frac{\varepsilon }{k_{4}}Ā\bar{T}\right)d_{4}>\frac{\varepsilon }{k_{4}}Ā\left|k_{4}-Ā\right|d_{2}+\delta d_{3}, \end{array}\right)$$

because *Ā *can be greater than *k*_4_. The equilibrium values correspond to the point ${\bar{Q}}^{c}$, given by equation (9).

Let us define

$$\left\{\begin{array}{c} d_{1}=\frac{\gamma k_{2}+\varphi }{\alpha_{2}}\\ d_{2}=1\\ d_{3}=\frac{\gamma^{2}k_{2}\bar{T}+\gamma \alpha_{2}Ē+\left(\gamma \bar{T}\alpha_{2}\right)\varphi }{\alpha_{2}\left(\mu_{4}+\delta \right)}\\ d_{4}=\frac{Ā-\varphi }{k_{3}-\bar{T}}, \end{array}\right)$$

where *φ *> 0. By these definitions, the first three inequalities of equation (B.1) hold. To prove the last inequality, we substitute above definitions, and we obtain

(B.2) $$0<\varphi <\frac{\varphi_{n}}{\varphi_{d}},$$

where the numerator *φ*_*n *_and denominator *φ*_*d *_*>*0 are

$$\left\{\begin{array}{c} \begin{array}{c} \varphi_{n}=\left(\delta \frac{\bar{P}}{Ā}+\frac{\varepsilon }{k_{4}}Ā\bar{T}\right)\frac{Ā}{k_{3}-\bar{T}}-\frac{\varepsilon }{k_{4}}Ā\left|k_{4}-Ā\right|\\ \;\;\;-\frac{\delta \gamma k_{2}\left(\alpha_{2}-\mu_{2}\right)}{\alpha_{2}\left(\mu_{4}+\delta \right)} \end{array}\\ \varphi_{d}=\left(\delta \frac{\bar{P}}{Ā}+\frac{\varepsilon }{k_{4}}Ā\bar{T}\right)\frac{1}{k_{3}-\bar{T}}+\frac{\delta \left(\gamma \bar{T}+\alpha_{2}\right)}{\alpha_{2}\left(\mu_{4}+\delta \right)}, \end{array}\right)$$

with the last term of the numerator being obtained using the relation

$$\gamma^{2}k_{2}\bar{T}+\gamma \alpha_{2}Ē=\gamma k_{2}\left(\alpha_{2}-\mu_{2}\right),$$

which is valid in the equilibrium. Since *φ*_*d *_> 0, if we show that *φ*_*n *_> 0, then there exists a positive number *φ*.

Let us consider the case where there are two positive solutions for the equation *f*(*T*) = *g*(*T*). In this case, we show that the bigger equilibrium ${\bar{Q}}_{>}^{c}$, with coordinate *T*_>_as solution of (5), is stable. Additionally, we assume that *Ā *>*k*_4_, which seems reasonable since the equilibrium ${\bar{Q}}^{c}$ is stable for $\bar{T}>\left(\alpha_{1}-\mu_{1}\right)/\beta_{1}=T_{C}$. Substituting the coordinates of the equilibrium ${\bar{Q}}^{c}$, the numerator of *φ *can be written as

$$\varphi_{n}=\frac{Ā}{{\left(k_{3}-\bar{T}\right)}^{2}}\Phi \left(\bar{T}\right),$$

with

(B.3) $$\begin{array}{ccc} \Phi \left(\bar{T}\right) & =\frac{\varepsilon }{k_{4}\alpha_{3}}\Phi_{1}\left(\bar{T}\right)-\frac{\gamma \delta k_{2}\alpha_{3}}{\alpha_{2}\left(\mu_{4}+\delta \right)\mu_{3}k_{3}} & \\ & \;\times {\left(k_{3}-\bar{T}\right)}^{2}\Phi_{2}\left(\bar{T}\right), \end{array}$$

where

$$\left\{\begin{array}{c} \begin{array}{c} \Phi_{1}\left(\bar{T}\right)=k_{4}\alpha_{3}{\bar{T}}^{2}-2k_{3}\left(k_{4}\alpha_{3}-\mu_{3}\right)\bar{T}\\ \;\;\;\;\;+k_{3}^{2}\left(k_{4}\alpha_{3}-\mu_{3}\right) \end{array}\\ \Phi_{2}\left(\bar{T}\right)=\gamma {\bar{T}}^{2}-2\left(\alpha_{2}-\mu_{2}\right)\bar{T}+\left(\alpha_{2}-\mu_{2}\right), \end{array}\right)$$

and we must show that $\Phi \left(\bar{T}\right)>0$, for higher value of $\bar{T}$, that is, $\bar{T}=T_{>}$. Let us assume that *k*_4_*α*_3 _>*μ*_3_. Then, we have $\Phi \left(\bar{T}\right)>0$, for $\bar{T}\geq 0$, because the discriminant of $\Phi_{1}\left(\bar{T}\right)$ is

$$\Delta_{1}=-4k_{3}^{2}\mu_{3}\left(k_{4}\alpha_{3}-\mu_{3}\right)<0.$$

Hence, $\Phi_{2}\left(\bar{T}\right)$ determines the existence of positive *φ*_*n*_. The discriminant of $\Phi_{2}\left(\bar{T}\right)$ is

$$\Delta_{2}=4_{\gamma }\left(\alpha_{2}-\mu_{2}\right)\left(T_{E}-T_{A}\right),$$

where *T*_*E *_and *T*_*A *_are given in equation (7). We have two possibilities. First, when *T*_*E *_<*T*_*A*_, we have $\Phi_{2}\left(\bar{T}\right)>0$, for all $\bar{T}\geq 0$, and $\Phi \left(\bar{T}\right)>0$ for

$$\varepsilon >\varepsilon_{\text{min}},$$

where

(B.4) $$\varepsilon_{\text{min}}=\frac{\gamma \delta k_{2}\alpha_{3}k_{4}\alpha_{3}}{\alpha_{2}\left(\mu_{4}+\delta \right)\mu_{3}k_{3}}{\left(k_{3}-T_{>}\right)}^{2}\frac{\Phi_{2}\left(T_{>}\right)}{\Phi_{1}\left(T_{>}\right)}.$$

Hence, when *ε *>*ε*_min_, *φ*_*n *_> 0 and we have a positive number *φ *obeying equation (B.2). Second, when $T_{E}>T_{A},\Phi_{2}\left(\bar{T}\right)$ has two positive solutions Φ_2*<*_and Φ_2*>*_given by

$$\left\{\begin{array}{c} \Phi_{2<}=\frac{\alpha_{2}-\mu_{2}}{\gamma }-\sqrt{\frac{\alpha_{2}-\mu_{2}}{\gamma }\left(\frac{\alpha_{2}-\mu_{2}}{\gamma }-k_{3}\right)}\\ \;\;\;\;=T_{E}-\sqrt{T_{E}\left(T_{E}-T_{A}\right)}\\ \Phi_{2>}=\frac{\alpha_{2}-\mu_{2}}{\gamma }+\sqrt{\frac{\alpha_{2}-\mu_{2}}{\gamma }\left(\frac{\alpha_{2}-\mu_{2}}{\gamma }-k_{3}\right)}\\ \;\;\;\;=T_{E}+\sqrt{T_{E}\left(T_{E}-T_{A}\right),} \end{array}\right)$$

and we have $\Phi_{2}\left(\bar{T}\right)\leq 0$, for $\Phi_{2<}\leq \bar{T}\geq \Phi_{2>}$; otherwise, $\Phi_{2}\left(\bar{T}\right)>0$. Notice that Φ_2>_is out of the range of feasibility, once Φ_2>_>*T*_*E*_. We know that $\left(\alpha_{1}-\mu_{1}\right)/\beta_{1}<\bar{T}<k_{3}$, but the bigger solution under consideration is $T_{>}=\left(\alpha_{1}-\mu_{1}\right)/\beta_{1}^{c}=T_{E}^{c}$, for $\beta_{1}\geq \beta_{1}^{c}$. It is easy to show that Φ_2_(*k*_3_) *<*0; but, $\Phi_{2}\left(T_{E}^{c}\right)\leq 0$, if $T_{E}^{c}\geq \Phi_{2<}$, and $\Phi_{2}\left(T_{E}^{c}\right)>0$, if $T_{E}^{c}<\Phi_{2<}$. Hence, $\Phi_{2}\left(\bar{T}\right)\leq 0$ if $T_{E}^{c}<\Phi_{2<}$. Hence, $\Phi \left(\bar{T}\right)>0$, for $\bar{T}\geq 0$, when $T_{E}^{c}\geq \Phi_{2<}$, or when $T_{E}^{c}\geq \Phi_{2<}$ and $\bar{T}\geq \Phi_{2<}$; and $\Phi \left(\bar{T}\right)>0$, when $T_{E}^{c}<\Phi_{2<}$ and $T_{E}^{c}<\bar{T}<\Phi_{2<}$ for *ε > ε*_min_.

Summarizing, *φ*_*n *_> 0 occurs, in order to have positive number *φ*:

**1**. *T*_*A *_<*T*_*E *_or *γ *< (*α*_2 _- *μ*_2_)/*k*_3 _- weak capacity of recruitment of the normal epithelial cells by cancer cells. We have:

**1.a ***If *$T_{E}^{c}\geq \Phi_{2<}:\varphi_{n}>0$ without restriction about *T*_>_.

**1.b **If $T_{E}^{c}<\bar{T}<\Phi_{2<}$: we have two possibilities

**1.b.1 **If *T*_>_*>*Φ_2*<*_: *φ*_*n *_> 0 without restriction about *T*_>_.

**1.b.2 **If *T*_>_*<*Φ_2*<*_: *φ*_*n *_> 0 if *ε > ε*_min_, where *T*_>_satisfies *ε*_min_, equation (B.4). Higher proliferation of angiogenesis cells must occur.

2. *T*_*A *_>*T*_*E *_or *γ *> (*α*_2 _- *μ*_2_)*/k*_3 _- strong capacity of coopting normal epithelial cells by cancer cells: *φ*_*n *_*>*0, if *ε *>*ε*_min_, where *T*_>_satisfies *ε*_min_.

When *γ *is small, the big equilibrium ${\bar{Q}}_{>}^{c}$, with one coordinate *T*_>_, is stable without conditions (cases 1.a and 1.b.1). However, for sufficiently higher values of *γ*, the big equilibrium point ${\bar{Q}}_{>}^{c}$ can be unstable (case 2), in which case Hopf bifurcation can occur (see Appendix C).

In the case of the small equilibrium ${\bar{Q}}_{<}^{c}$, with one coordinate *T*_<_, it is unstable. Assuming that *Ā < k*_4_, we show here that *A *corresponding to small *T*_<_is not an *M*-matrix (we are not proving that ${\bar{Q}}_{<}^{c}$ is unstable). In this case, equation (B.3) becomes

$$\begin{array}{ll} \Phi \left(\bar{T}\right) & =-\frac{\varepsilon }{k_{4}\alpha_{3}}\Phi_{1}\left(\bar{T}\right)-\frac{\gamma \delta k_{2}\alpha_{3}}{\alpha_{2}\left(\mu_{4}+\delta \right)\mu_{3}k_{3}}\;\\ & \;\times {\left(k_{3}-\bar{T}\right)}^{2}\Phi_{2}\left(\bar{T}\right),\;\\ & \; \end{array}$$

where

$$\left\{\begin{array}{c} \Phi_{1}\left(\bar{T}\right)=k_{4}\alpha_{3}{\left(\bar{T}-k_{3}\right)}^{2}-\mu_{3}k_{3}^{2}\\ \Phi_{2}\left(\bar{T}\right)=\gamma {\bar{T}}^{2}-2\left(\alpha_{2}-\mu_{2}\right)\bar{T}+\left(\alpha_{2}-\mu_{2}\right). \end{array}\right)$$

The bigger roots of $\Phi_{1}\left(\bar{T}\right)$ and $\Phi_{2}\left(\bar{T}\right)$ are not biologically feasible, because they are, respectively, higher than *k*_3 _and (*α*_2 _- *μ*_2_)/*γ*. Let us define $T_{m}=\text{min}\left\{\overset{1}{\underset{<}{T}},T_{<}^{2}\right\}$, where $\text{min}\left\{\overset{1}{\underset{<}{T}},T_{<}^{2}\right\}$ is the minimum between the small roots of, respectively, $\Phi_{1}\left(\bar{T}\right)$ and $\Phi_{2}\left(\bar{T}\right)$ given by $T_{<}^{1}=k_{3}\left(1-\sqrt{1-\frac{\mu_{3}}{k_{4}\alpha_{3}}}\right)$, assuming that *k*_4 _≥ 1, and $T_{<}^{2}=T_{E}\left(1-\sqrt{1-\frac{T_{A}}{T_{E}}}\right)$, assuming that *T*_*E *_≥ *T*_*A*_. Hence, if $\bar{T}<T_{m}$, we have $\Phi \left(\bar{T}\right)>0$ and there is not a positive number *φ*. Notice that, when *T*_*E *_<*T*_*A*_, we have $\Phi_{2}\left(\bar{T}\right)>0$, and it is enough to satisfy $\bar{T}<T_{<}^{1}$.

## Appendix C: Hopf bifurcation

**Figure 19:**
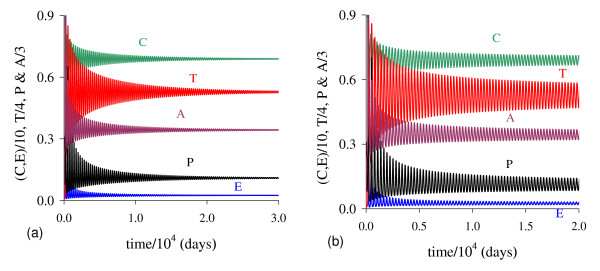
**Dynamical trajectories using *γ *near $\gamma_{1}^{c}$**. Dynamical trajectories of the system (1) considering the values of parameters given in Table 2, except *γ *near $\gamma_{1}^{c}$. When *T*(0) = 0.45 (for *T*(0) = 0.44 both cases go to ${\bar{Q}}^{0}$), ${\bar{Q}}_{>}^{*}$ is the attracting point for *γ *= 2.31 *× *10^-2 ^(a), and limit cycle with small amplitude circulating unstable ${\bar{Q}}_{>}^{*}$ appears for *γ *= 2.32 *× *10^-2 ^(b). The scales of vertical and horizontal axes must be multiplied by the factors shown in the legends to obtain the actual values.

**Figure 20:**
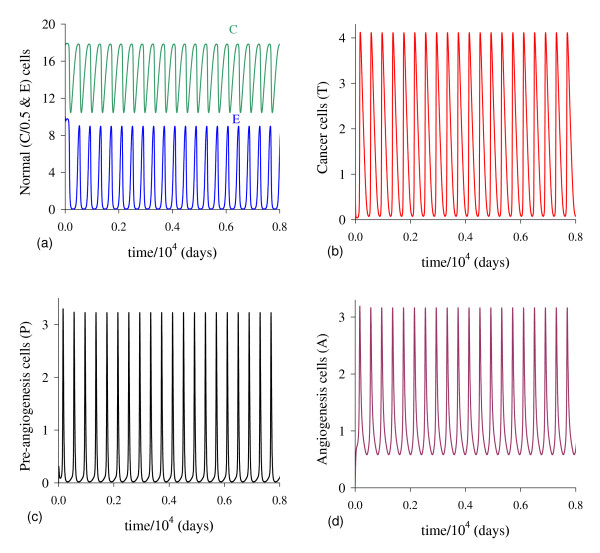
**Dynamical trajectories using *γ *near but lower than $\gamma_{2}^{c}$**. Dynamical trajectories of the system (1) considering the values of parameters given in Table 2, except *γ *near but lower than $\gamma_{2}^{c}$. When *T*(0) = 0.42 (${\bar{Q}}^{0}$ is attracting for *T*(0) = 0.41), limit cycle with large amplitude circulating unstable ${\bar{Q}}_{>}^{*}$ occurs for *γ *= 2.519 *× *10^-2^. Regular oscillations are observed in all variables: *C *and *E *(a), *T *(b), *P *(c) and *A *(d). The scales of vertical and horizontal axes must be multiplied by the factors shown in the legends to obtain the actual values.

**Figure 21:**
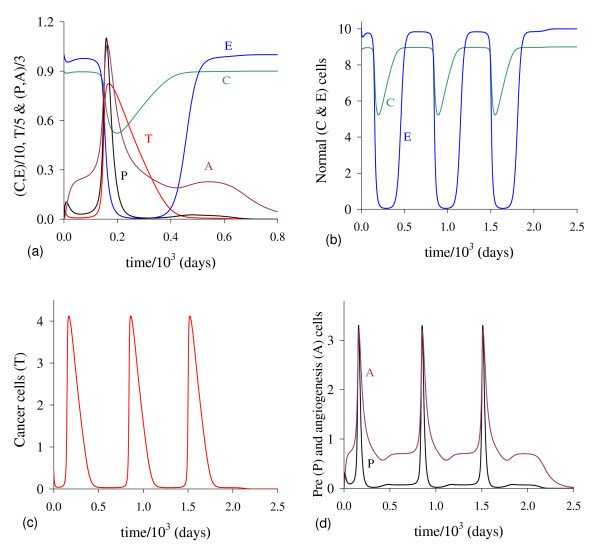
**Dynamical trajectories using *γ *near but greater than $\gamma_{2}^{c}$**. Dynamical trajectories of the system (1) considering the values of parameters given in Table 2, except *γ *near but greater than $\gamma_{2}^{c}$. When *T*(0) = 0.42 (${\bar{Q}}^{0}$ is attracting for *T*(0) = 0.41), limit cycle disappears for *γ *= 2.520 *× *10^-2^. Being ${\bar{Q}}_{>}^{*}$ unstable, the dynamical trajectories go to trivial ${\bar{Q}}^{0}$ after one oscillation (a). However, the number of oscillations increases if *γ *is very close to $\gamma_{2}^{c}$. For *γ *= 2.519675 *× *10^-2 ^and *T*(0) = 0.42, ${\bar{Q}}^{0}$ is attained after three oscillations: *C *and *E *(b), *T *(c), *P *and *A *(d). The scales of vertical and horizontal axes must be multiplied by the factors shown in the legends to obtain the actual values.

In Figures 19, 20 and 21 we illustrate the Hopf bifurcation (see Figure 10 in the main text), using values of parameters given in Table 2, except *γ *assuming higher values. The following figures are mathematical results, not cancer in an organ.

Dynamical trajectories are shown in Figure 19 for *γ *near $\gamma_{1}^{c}:\gamma_{1}=2.31\times 10^{-2}≲\gamma_{c}^{1}$ and $\gamma_{2}=2.32\times 10^{-2}≳10^{-2}≳\gamma_{1}^{c}$. When *T*(0) = 0.44, trajectories of both cases go to ${\bar{Q}}^{0}$ (trajectories similar to Figure 1(a), not shown). When *T*(0) = 0.45, dynamical trajectories for *γ*_1 _go to stable ${\bar{Q}}_{>}^{*}$ (a), while for *γ*_2_, they oscillate around unstable ${\bar{Q}}_{>}^{*}$ (b), in which case limit cycle arises.

Now, we show the dynamical trajectories for *γ *near $\gamma_{2}^{c}$. Again, for $\gamma_{3}=2.519\times 10^{-2}≲\gamma_{2}^{c}$ and $\gamma_{4}=2.520\times 10^{-2}≳\gamma_{2}^{c}$, when *T*(0) = 0.41, trajectories of both cases go to ${\bar{Q}}^{0}$ (trajectories similar to Figure 1(a), not shown). When *T*(0) = 0.42, Figure 20 shows dynamical trajectories for *γ*_3 _oscillating around unstable ${\bar{Q}}_{>}^{*}$. Notice that the amplitude of regular oscillations of the variables is very high: *C *and *E *(a), *T *(b), *P *(c) and *A *(d). For the same *T*(0) = 0.42, we show the dynamical trajectories for *γ*_4 _and *γ*_5 _= 2.519675 *× *10^-2^, which is slightly higher than $\gamma_{2}^{c}$. In both cases we have $\gamma >\gamma_{2}^{c}$, and Figure 21 shows trajectories going to ${\bar{Q}}^{0}$ with different number of oscillations: for *γ*_4 _(a), we have one oscillation, while for *γ*_5_, three oscillations, showing *C *and *E *(b), *T *(c), *P *and *A *(d). When $\gamma >\gamma_{2}^{c}$, we observe that *E *goes initially to zero, and, after 400 *days*, increases abruptly, according to Figure 21(a). In Figure 5(a) we showed that *C *has similar behavior.

Comparing Figures 19, 20 and 21, we observe that, as *γ *increases, the real part of the complex eigenvalues decreases (see Table 3), and damped oscillations persist for longer times. At $\gamma =\gamma_{1}^{c}$, real part is zero. For instance, a pair of complex number has real part -2.3 × 10^-7 ^at *γ *= 2.3196 *× *10^-2^, and +9.0 × 10^-7 ^at *γ *= 2.3197 × 10^-2^. In both cases, two of them are complex number with negative real part and one negative number. As *γ *increases sinceafter $\gamma_{1}^{c}$, amplitude of the limit cycle increases, and at $\gamma =\gamma_{2}^{c}$ disappears. Observe that a finite number of oscillations occurs before reaching the trivial equilibrium. The increasing in the amplitude of regular oscillations resulted in an unsustainable value of *T*_>_, and, hence, this value (due to being lower than a critical value) can not trigger new burst of cancer cells.

Numerical results regarding to *γ *showed that there is a threshold for *γ*, plus two critical values. For small values, *γ *<*γ*^*th*^, cancer cells can not induce new blood vessels, and cancer fades out. As *γ *increases, amplitude of damped oscillations increases and appears stable limit cycle. The limit cycle separates cancer state (${\bar{Q}}^{*}$) to cure (${\bar{Q}}^{0}$). However, the cure occurs at the expense of death of cancer cells due to elimination of the pre-existing network of blood vessels. This phenomenon occurs due to the absence of dependency between normal cells (*C*) and pre-existing epithelial cells (*E*) in the carrying capacities *k*_1 _and *k*_2_.
